# Advancing the WHO-INTEGRATE Framework as a Tool for Evidence-Informed, Deliberative Decision-Making Processes: Exploring the Views of Developers and Users of WHO Guidelines

**DOI:** 10.34172/ijhpm.2020.193

**Published:** 2020-10-27

**Authors:** Jan M. Stratil, Deepak Paudel, Karen E. Setty, Carlos E. Menezes de Rezende, Aline A. Monroe, Jimmy Osuret, Inger B. Scheel, Manfred Wildner, Eva A. Rehfuess

**Affiliations:** ^1^Institute for Medical Information Processing, Biometry, and Epidemiology – IBE, LMU Munich, Munich, Germany.; ^2^Pettenkofer School of Public Health, Munich, Germany.; ^3^Save the Children, Kathmandu, Nepal.; ^4^The Water Institute, Department of Environmental Sciences and Engineering, The University of North Carolina at Chapel Hill, Chapel Hill, NC, USA.; ^5^National Supplementary Health Agency, Ministry of Health, Brasília, Brazil.; ^6^Department of MaternalInfant Nursing and Public Health, College of Nursing, University of São Paulo, Ribeirão Preto, Brazil.; ^7^Department of Disease Control and Environmental Health, Makerere University School of Public Health, Kampala, Uganda.; ^8^Department of Global Health, Norwegian Institute of Public Health, Oslo, Norway.; ^9^Bavarian Health and Food Safety Authority, Munich, Germany.

**Keywords:** Decision-Making, Priority Setting, Resource Allocation, Guideline Development, World Health Organization, Framework

## Abstract

**Background: **Decision-making on matters of public health and health policy is a deeply value-laden process. The World Health Organization (WHO)-INTEGRATE framework was proposed as a new evidence-to-decision (EtD) framework to support guideline development from a complexity perspective, notably in relation to public health and health system interventions, and with a foundation in WHO norms and values. This study was conducted as part of the development of the framework to assess its comprehensiveness and usefulness for public health and health policy decision-making.

**Methods:** We conducted a qualitative study comprising nine key informant interviews (KIIs) with experts involved in WHO guideline development and four focus group discussions (FGDs) with a total of forty health decision-makers from Brazil, Germany, Nepal and Uganda. Transcripts were analyzed using MAXQDA12 and qualitative content analysis.

**Results: **Most key informants and participants in the FGDs appreciated the framework for its relevance to real-world decision-making on four widely differing health topics. They praised its broad perspective and comprehensiveness with respect to new or expanded criteria, notably regarding societal implications, equity considerations, and acceptability. Some guideline developers questioned the value of the framework beyond current practice and were concerned with the complexity of applying such a broad range of criteria in guideline development processes. Participants made concrete suggestions for improving the wording and definitions of criteria as well as their grouping, for covering missing aspects, and for addressing overlap between criteria.

**Conclusion: **The framework was well-received by health decision-makers as well as the developers of WHO guidelines and appears to capture all relevant considerations discussed in four distinct real-world decision processes that took place on four different continents. Guidance is needed on how to apply the framework in guideline processes that are both transparent and participatory. A set of suggestions for improvement provides a valuable starting point for advancing the framework towards version 2.0.

## Background

**Key Messages**

** Implications for policy makers**

- Public health and health policy processes are complex and deeply value-laden. This includes different types of decision-making processes at national or sub-national levels as well as the development of guidelines at a global level.
- The various affected stakeholder groups have their own reasons and principles guiding their decisions. Involving diverse stakeholders and taking their views into account in a structured way can ensure transparency, legitimacy, and acceptability of the decision, and increase the likelihood of implementation. Evidence-to-Decision (EtD) frameworks, such as the World Health Organization (WHO)-INTEGRATE framework, can serve as helpful tools.
- The WHO-INTEGRATE framework could be a valuable tool to support decision-making processes and, with regard to WHO guideline development, could enhance the relevance and applicability of WHO recommendations in public health and health policy. Suggestions provided will help to further advance the framework and to develop concrete guidance on how to apply it in practice.

** Implications for the public**

When making public health or health policy decisions, decision-makers should consider the best available scientific evidence and other factors (eg, cost, feasibility, or acceptability). They should also ensure that members in the committee preparing or making decisions is sufficiently diverse and represents all relevant viewpoints. This applies to political decisions at national or subnational levels and to more technical processes, eg, development of guidelines. When not adequately or transparently considered, decisions may not lead to the desired impacts or may not be considered acceptable and legitimate. Decision frameworks, such as the World Health Organization (WHO)-INTEGRATE framework, can support decision-makers and help ensure that all factors of relevance are considered. We discussed this framework with developers of guidelines at the WHO and with groups of decision-makers across four continents. They reported that the factors (called criteria) included in this framework are both comprehensive and relevant to real world public health and health policy decisions. This suggests that the WHO-INTEGRATE framework can be a valuable tool for application from global to local levels.

Making evidence-informed decisions about public health and health system interventions and policies is complex.^1-3^ On the one hand, producing and assessing evidence eg, on the effectiveness of public health and health policy interventions is challenging due to the complexity of the interventions themselves (eg, the number of components, or the pathway leading to multiple outcomes).^4^ Furthermore, due to interactions with the system in which these are implemented (eg, system changes due to emergent properties, adaptivity, or feedback mechanisms) as well as due to the high context-dependency of the effects of the intervention.^2,5^ On the other hand, simply producing more and stronger evidence eg, on the efficacy or cost-effectiveness of an intervention is in itself not sufficient to make better choices, as evidence-informed decision-making is a deeply value-laden process.^6-8^ Decision-makers must balance numerous and often conflicting normative and technical aspects for a decision-making process,^9-11^ which represents an additional source of complexity (eg, which criteria should be considered and how should these be weighed against each other)? This holds true for all forms of structured decision-making processes in health, notably priority setting,^6,12,13^ health technology assessments,^14^ and the development of guidelines.^15,16^

 To support decision-makers in making informed decisions on matters of public health and health policy, the World Health Organization (WHO) provides systematically developed guidelines.^15^ The recommendations set forth in these guidelines are particularly important for policy-makers and program managers in low- and middle-income countries who often have limited resources for conducting comprehensive processes of evidence gathering and analysis. Those responsible for developing WHO guidelines are challenged to balance the need for a comprehensive approach – which is indicated due to the complexity of the intervention, the challenges of evidence generation, and the multiplicity of values affected – with the necessity to provide recommendations in a timely manner and often under considerable resource constraints.

 In guidelines and beyond, various approaches to integrate a range of specific considerations have been proposed. These approaches – the Accountability for Reasonableness (A4R) framework by Daniels and Sabin^17-21^ among others^6,14,22,23^ – emphasize the importance of transparency throughout the process, the inclusion of relevant stakeholders, the appropriate composition of the decision-making panel, and the identification and weighting of criteria to be considered, as well as the possibility of revisions and appeals. According to A4R,^17^ one key aspect is the condition of relevance: the decision or recommendation must rest on evidence, reasons, and principles that all fair-minded parties can agree are relevant to deciding how to meet the diverse needs of affected stakeholders under the imposed resource constraints.^17^ Such processes can increase the acceptability and perceived legitimacy of a decision^19,24,25^ even if – given varying and sometimes contradictory interests – no consensus regarding the *right* selection and weighting of criteria can be achieved.^19^

 Involving representatives of all relevant stakeholder groups, including community representatives (eg, citizens, patients) in the process of identifying these reasons and principles is considered ideal.^6,14,26,27^ However, it is often difficult to meet this ideal due to time and resource constraints; this increases the risk of relevant criteria being overlooked. While not intended to nor able to replace stakeholder participation, Evidence-to-Decision (EtD) frameworks can support decision-makers and guideline developers in this balancing act.^28,29^ EtD frameworks tend to comprise sets of criteria as well as procedural guidance. They are intended to ensure that all relevant criteria are considered, the best available evidence is assessed, and the underlying rationale is made explicit and transparent.^30^ When applied well, these EtD frameworks can help identify and integrate the criteria of relevance for a given decision-making process, even if the voices of all relevant stakeholders were not heard. Their use should, however, not be misinterpreted as a justification for an unbalanced or incomplete composition of the committee preparing or making decisions. Furthermore, structured processes guided by EtD frameworks can lead to better, more rational decisions by counteracting inadequate (cognitive, emotional or social) heuristics, cognitive biases, or in-group dynamics.^31-35^ Therefore, EtD frameworks should be as comprehensive as possible, which often is at odds with the constraints, and needs to be balanced against the resources and time available for developing an informed decision.

 WHO uses EtD frameworks in their process to develop guidelines.^15,27^ Given the reach and potential impact of the recommendations set forth in WHO guidelines, the nature of the guideline development process and the criteria used to inform recommendations set a benchmark for other uses. Both are described in the WHO guideline handbook for guideline development.^27^ In formulating recommendations WHO Guideline Development Groups (GDG)^27^ are asked to consider not only evidence of effectiveness but also a range of other criteria (eg, resource implications, acceptability, feasibility^27^) derived from an early version of the Grading of Recommendations Assessment, Development and Evaluation (GRADE) EtD framework.^27,30,35^ Methods for guideline development were originally tailored to clinical interventions, and are still profoundly influenced by this field.^36,37^ WHO recently commissioned a series of papers to make guideline development methods more applicable to complex public health and health system challenges.^15^ In this context, a new EtD framework, the WHO-INTEGRATE framework version 1.0,^16^ was developed with a strong conceptual and normative foundation^16^ primarily based on an evaluation of WHO norms and values derived from key WHO documents (eg, the WHO constitution) and widely used public health ethics frameworks. To ensure the relevance of the framework, this normative approach was combined with a literature review of decision criteria used in real world decision-making,^38^ and an assessment of complexity features.^16^

 The WHO-INTEGRATE framework comprises six criteria – *balance of health benefits and harms*; *human rights and sociocultural acceptability*; *health equity*, *equality and non-discrimination*; *societal implications; financial and economic considerations*; and *feasibility and health system considerations *– as well as the meta-criterion *quality of evidence*. Each criterion encompasses a detailed definition, a set of sub-criteria, example questions to assess these sub-criteria and a methodological toolbox with suggested methods for collecting, synthesizing and appraising evidence. Applicability and benefit of the WHO-INTEGRATE framework to real-world decision-making situations remain to be tested.

 The objective of this study was to assess the WHO-INTEGRATE framework version 1.0 through a wider participatory process with experts involved in developing WHO guidelines on an international level as well as decision-makers developing national guidelines and/or adapting and implementing WHO guidelines on a national level. Specifically, it served to review the framework in terms of its overall structure and specific criteria and to shed light on the comprehensiveness, relevance, and usefulness in real-world decision-making contexts.

## Methods

 We conducted nine key informant interviews (KIIs) with experts involved in WHO guideline development on an international level as well as four focus group discussions (FGDs) with health decision-makers in Brazil, Germany, Nepal, and Uganda.

###  Participants and Data Collection

####  Key Informant Interviews

 The KIIs were conducted with experts who had recently participated in a WHO guideline development process, either as the coordinating WHO staff, GDG chair, or the methodologist supporting the process. In consultation with the Secretariat of the WHO Guideline Review Committee, three WHO guidelines – on sexual and reproductive health and rights of women living with HIV,^39^ on communicating risk in public health emergencies^40^ and, on antenatal care for a positive pregnancy experience^41^ – were selected purposefully, with the aim to cover distinct types of interventions and to capture positive as well as challenging experiences with applying the GRADE EtD framework.^27^

 In the face-to-face or telephone/video interviews carried out between June and October 2017, we used a semi-structured, pre-tested interview guide, developed based on the guiding research questions (Supplementary file 1). The first interview part focused on the experience of using the GRADE EtD framework to formulate recommendations and decide on their strength. In the second part, the interviewees received an interim version of the WHO-INTEGRATE framework and were asked to comment on practical considerations (eg, understandability), the framework content (eg, missing criteria), and the implications of using the framework in WHO guideline development processes.

 The audio files of the recorded interviews were transcribed, reviewed, pseudonymized, and then deleted.

####  Focus Group Discussions

 The FGDs were conducted with decision-makers across four countries and continents, as detailed in Table 1 and Supplementary file 2. To capture the diversity of views, we sought to maximize heterogeneity among countries (ie, country income group, region) and topics of discussion (ie, type of intervention/approach).

**Table 1:** Countries, Thematic Areas and Topics of FGDs

**Country**	**Brazil**	**Germany**	**Nepal**	**Uganda**
Thematic area	Infectious diseases; healthcare system	Public health nutrition; non-communicable diseases	Sexual and reproductive health; health services research	WaSH; infectious diseases
Topic of FGD	Tuberculosis guidelines and decentralized actions related to tuberculosis control	(Health) implications of an elimination of an EU quota system on isoglucose and considerations regarding countermeasures (eg, labelling, taxation and/or prohibition of products)	Health services related to sexual reproductive health and rights of adolescents	Management of untreated wastewater, including sewage from septic tanks and fecal sludge from pit latrines
Country income group	Middle-income country	High-income country	Low-income country	Low-income country
WHO region	Latin American Region	European Region	South East Asian Region	African Region
Researcher(s) conducting FGD	AAM, CEMR	JMS	DP	JO, KS
Date of FGD	June 2018	June 2018	October 2017	August 2017
Number of participants	n = 17	n = 7	n = 8	n = 8
Characteristics of participants and rationale	Multidisciplinary staff and invited members of National Coordination for Tuberculosis Control Program directly dealing with national policies implementation, decision-making and public health protocols design and adaptation	Staff of the Bavarian Health and Food Safety Authority across several departments advising on and preparing decisions regarding food safety and food regulation on the level of a German federal state	National level experts from governmental institutions (eg, divisions of ministry of health) and Nepali experts from national and international NGOs working on development and implementation of programs of and rights ASRH	Members of a national workgroup on water and sanitation from diverse organizational sectors, including national and sub-national government, nongovernmental/civil society, NGO network, and private actors with expertise in water and sanitation guideline implementation; including Ugandan representative of the Sanitation and Water for All network and WHO sanitation guideline development; (note: foreign aid and multilateral organizations were excluded)
Recruitment approach	Direct contact with implementation science expert and researchers working on the topic to identify a diverse set of key experts; invitation of identified experts by the local researchers directly via email.	Direct contact (personal, via telephone or email) of staff members involved with analyzing the implications of food safety or providing guidance on such countermeasures within the Bavarian Health and Food Safety Authority	Direct contact (in person, telephone) to individual experts from the federal ministry of health and individual national and international NGOs; snowballing recruitment of additional experts in the field through the directly contacted experts	In-person recruitment of key contacts at professional conferences followed by more extensive email recruitment within national water and sanitation work group; snowballing recruitment through referral to other potential participants in their networks of professionals doing work on national WaSH issues
Duration FGD	130 minutes	95 minutes	100 minutes	120 minutes
Setting of data collection	Brasília, Brazil Conference room in the building of the National Coordination for the National Tuberculosis Control Program	Munich, Germany Conference room in the building of the Bavarian Health and Food Safety Authority (1 participant via video link)	Kathmandu, Nepal Conference room at centrally located hotel	Kampala, Uganda Conference room at WHO offices
Language of FGD/analysis	Portuguese/English	German/German	Nepali/English	English/English

Abbreviations: WaSH, Water, sanitation, and hygiene; FGD, focus group discussion; EU, European Union; WHO, World Health Organization; ASRH, adolescent sexual and reproductive health and rights; NGO, non-governmental organization.

 Local researchers undertook the FGDs in close collaboration with the developers of the framework between August 2017 and October 2018. A topic of current importance was suggested by the local researchers. Decision-makers were identified by the local contact, who reached out to a purposive sample of experts involved with making national recommendations, and potential WHO guideline users, ie, those responsible for adapting and implementing recommendations locally, regionally, or nationally. The recruitment strategy and composition of decision-makers varied across the four FGDs regarding the nature of the committee (providing advice vs. making recommendations), the level of decision-making (national vs. local) and exact composition (representative sub-set of committee vs. ad hoc assembly of participants) (see Table 1 for details).

 We developed a preliminary interview guide based on the KII interview guide and adapted it to the setting and topic of the FGDs (Supplementary file 1). The FGDs were set up as a thought experiment: First, the participants conducted a guided brainstorming session on criteria of relevance for the decision-making process on their chosen topic, at which point they were unaware about the content of the WHO-INTEGRATE framework. This was one in order to not have the discussion of criteria and considerations of relevance be “contaminated” or framed by the content of the framework. Second, they were presented with an interim version of the framework and asked to review whether the framework covered the previously discussed criteria, whether aspects were missing, and whether there might be specific suggestions for improvement.

 The files of the audio-recorded FGDs were transcribed and the transcripts reviewed by the local researchers. Transcripts were subsequently pseudonymized, translated into English (Brazil and Nepal) and reviewed after translation in the light of the audio-records. Audio-records were then deleted.

###  Data Analysis

 A two-person team (JMS and IS, ST or KK) analyzed the pseudonymized transcripts (KIIs and FGDs) through qualitative content analysis, following the approach by Mayring.^42^ Findings for the FGDs were provided to local contacts for feedback, clarification, and discussion. The analysis of the transcript of the FGD conducted in Germany was not translated but rather analyzed in German by two native speakers (JMS and KK). We followed mixed deductive and inductive approaches to develop the coding frames (Supplementary file 3) using the software MAXQDA12 (VERBI Software GmbH, Berlin). Furthermore, one researcher (JMS) assessed coded passages of the FGD transcripts with respect to whether aspects considered relevant for decision-making on the topic and theme in question were covered by the criteria and sub-criteria of the WHO-INTEGRATE framework.

## Results

 We conducted nine KIIs with WHO guideline staff (n = 2), GDG chairs (n = 4), and methodologists (n = 3) with a median duration of 62 minutes (range 57-69 minutes). Two additionally intended interviews did not take place, as one participant had retired (and therefore declined) and one interview could not be scheduled despite repeated attempts.

 The duration of the four FGDs with between seven and seventeen participants ranged from 95-150 minutes (Table 1, Supplementary file 2). The topics were: tuberculosis guidelines and decentralized actions related to tuberculosis control (Brazil), the (health) effects of an increase of isoglucose in food and potential countermeasures (Germany), health services related to sexual reproductive health and rights of adolescents (Nepal), and the management of untreated wastewater, including sewage from septic tanks and fecal sludge from pit latrines (Uganda).

###  Overview of Focus Group Discussions

 For conciseness, we aim to provide a synthesis rather than detailed account of all four FGDs here. Supplemental data is available by contacting the authors. The next section outlines the first phase of the discussions, as this varied, and summarizes selected main themes.

####  Focus Group Discussion in Brazil

 The FGD in Brazil was concerned with the development of tuberculosis guidelines and manuals within the Brazilian national plan to fight tuberculosis.

 During the first phase, participants discussed their experiences with the development of this plan, covering both general challenges in guideline development (eg, alignment with national and supranational strategies) and specific considerations in making recommendations; most often they referred to testing strategies as an example. Criteria of relevance brought up during this phase included affordability, availability, acceptability of healthcare access, adherence to treatments, economic and financial feasibility, cost-effectiveness, and political importance.

 A central consensual theme was the need to accommodate the realities of a heterogeneous country (“multiple Brazils”) comprising municipalities with high- versus low-disease burdens, and the related needs to address subsidiarity and empowerment of municipalities to develop locally adapted approaches and to target social determinants within a health in all policies approach.

 A second major theme was the criterion of acceptability: participants agreed on the importance of socio-cultural acceptability, especially among those intended to implement the intervention and the intended beneficiaries, stressing that acceptability could vary greatly even on a local level (eg, between healthcare institutions) and across population groups (eg, across different ethnicities or religious groups). Participants thought it unlikely to achieve socio-cultural acceptability overall. Several stated that they were unsure about how to handle the acceptability criterion based on a lack of or false knowledge among those rejecting an intervention (eg, the belief that a vaccination was developed to kill elderly).

 An important point of controversy related to the question whether a separate sub-criterion regarding the right to health needed to be added. The argument in favour of adding a sub-criterion was the framework’s focus on health, the argument against doing so was that the right to health is already covered within the broader sub-criterion on human rights.

####  Focus Group Discussion in Germany

 The FGD in Germany focused on food safety and food regulation, notably the expiration of an quota system of the European Union (EU) on the market share of isoglucose, which is expected to increase high-fructose corn syrup (HFCS) in foods and beverages. Due to concerns about adverse health effects of HFCS among the general public and parliamentarians, participants discussed whether the Bavarian Health and Food Safety Authority should issue a recommendation on countermeasures.

 During a first phase, participants were presented with a rapid literature review, concluding that there is an ongoing controversy regarding adverse health effects of HFCS. The participants then engaged in lively discussions on whether countermeasures such as labelling, taxation and/or prohibition of products should be taken.

 A central theme in the debate was the legal feasibility of such measures and lack of clarity regarding the responsible political level (ie, federal state, nation state, EU): participants discussed whether it would be in line with EU and national regulations and law if the Bavarian government would adopt and implement regulations.

 A major point of controversy was the need to balance the expected avoidance of harm, the (lack of) certainty of evidence, and the intrusiveness of the intervention. Some participants argued that in view of inconclusive evidence of harm the government does not have a mandate to act. Others argued with the precautionary principle, which allows enacting regulations to protect public health despite unresolved uncertainties.

 A further controversy was on the role of evidence in decision-making: methodological and ethical challenges can be prohibitive in proving harm beyond a reasonable doubt. Conflicts of interest can distort the evidence, both with respect to industry-funded research and evidence generated by researchers with ideological or personal interests (white hat bias^43^).

####  Focus Group Discussion in Nepal

 The FGD in Nepal focused on adolescent sexual and reproductive health and rights (ASRH). During the first phase, participants voiced their views on important criteria for developing and implementing guidelines focused on ASRH. These included: the capacity of healthcare providers, privacy, and user-friendliness. The discussion then shifted towards experience with adapting international guidelines (eg, from WHO) and implementing ASRH programs. While international guidelines were considered useful for procuring resources from the government, the group agreed that simply transferring global guideline recommendations to local realities can be challenging (eg, due to limited acceptability or resources).

 A major topic of discussion was the issue of socio-cultural acceptability. Importantly, guideline recommendations and programs cannot achieve their intended goals if they do not meet the needs and expectations of adolescents. Participants suggested that this could be achieved by engaging adolescents in developing guidelines and thus creating ownership of the program. Acceptability also encompassed community norms and values, eg, regarding gender issues. If these are not considered, a program’s effectiveness and implementation would suffer.

 Another central theme was the need to take local realities into account with respect to feasibility considerations. This covers locally available infrastructure as well as financial, technical and human resources. Local coordination within and beyond the health sector was regarded as essential.

####  Focus Group Discussion in Uganda

 The FGD in Uganda focused on the management of untreated wastewater, including sewage from septic tanks and fecal sludge from pit latrines within the larger context of the WHO guidelines on sanitation and health.

 During the first phase, participants discussed the importance of managing wastewater, septage and sludge, as well as the reasons for developing (international) guidelines on the topic and for potentially opposing such guidelines. Participants brought up considerations with respect to health implications of the measure, implementation and maintenance costs, and feasibility considerations.

 A central theme was the interlinkage between financial costs, resource availability, feasibility, and acceptability of the intervention. Participants agreed that these aspects needed to be reflected both from the perspective of the national as well as the local government and end users. Guideline recommendations might be rejected if they were regarded as too imposing in terms of cost and resource claims or regarded as unachievable under local circumstances.

 A related central topic was the need to consider resource requirements broadly in guideline recommendations: These need to reflect the required institutional infrastructure beyond the immediate needs for implementing and maintaining the intervention, such as infrastructure for planning, budgeting, or procuring resources as well as monitoring and evaluation.

####  Differences and Commonalities Across the FGDs 

 Health implications of interventions were discussed in all four FGDs, as was the need for multisectoral collaboration, implications for the health system, and consequences beyond the health sector.

 While all FGDs emphasized the importance of evidence (regarding effectiveness), the debate in Germany focused on the trustworthiness of the evidence, while the other FGDs emphasized transferability and generalizability from an international level to local realities.

 Socio-cultural acceptability of the intervention was discussed across all FGDs but the focus on different stakeholder groups varied: the FGD in Nepal concentrated on intended beneficiaries and the general population, the FGDs in Brazil and Uganda discussed the acceptability among those implementing and those intended to benefit from the intervention, and the German FGD was focused on political acceptability.

 Furthermore, the need to consider local perspectives was raised in all FGDs. In Brazil, discussions primarily regarded the different needs and realities of municipalities, in Uganda and Nepal this focus lay on acceptability, resources availability, and feasibility, and in Germany with a concern was legal feasibility (of passing laws on the level of federal states). In this context, participants in the FGDs in Brazil, Nepal and Uganda emphasized procedural considerations in guideline development, highlighting the need to involve affected stakeholders in the process.

 The importance of international guidelines and recommendations (eg, from WHO) were discussed in the FGDs in Brazil, Nepal, and Uganda, eg, regarding their usefulness in developing local guidelines or gaining political support. These were not addressed in the German FGD.

 In Nepal and Uganda, there was limited controversy: FGD participants seemed to strive for consensus, eg, on regarding missing criteria. In contrast, within the FGD in Germany and, to a lesser extent, in Brazil, discussions were more controversial. To some degree this may be due to cultural norms (eg, regarding deference to authority or conflict tolerance).

###  General Reception of the WHO-INTEGRATE Framework 

 The majority of interviewees in the KIIs made positive remarks about the framework, notably its usefulness and comprehensiveness. One participant remarked that the new framework covers many important issues and expressed a clear preference for this framework compared to the one in the WHO guideline handbook.^27^ Several interviewees explicitly stated that the criteria covered in the framework are important and none could or should be dropped in order to reduce the workload. Explicit positive statements were made regarding the new or expanded criteria *Societal implications, Balance of health benefits and harms, Equity, equality and non-discrimination, Human rights and socio-cultural acceptability, *and* Feasibility and health system considerations* and their sub-criteria, for example:

*“I think that the framework in its new form with this additional guidance is really informative, and useful, and helpful to participants in these panels and hopefully leads to good recommendations” *(KII_Methodologist).

 One interviewee felt the WHO-INTEGRATE framework did not go far enough by following the same approach as the GRADE EtD framework, namely starting with a defined intervention, gathering evidence and deciding whether a recommendation should be made; accordingly, a more appropriate approach might be to focus on beneficiaries and ask what should be done to improve health and well-being. Another interviewee remarked that focusing on high quality, quantitative evidence of effectiveness for a clearly defined intervention and outcome may not be feasible for complex interventions.

 It was also noted that many aspects in the framework were highly context-dependent and may therefore be less applicable in the development of global guidelines. Furthermore, two interviewees questioned the added value beyond current practice and implied that all newly added sub-criteria could also be addressed as part of the GRADE EtD framework. One interviewee, however, recognized that more explicit sub-criteria could function as “signposts” for less experienced methodologists:

*“I like the idea of making it more explicit so that you do think of these things. But if you’re quite a high-level expert, you would automatically do that […]” *(KII_Methodologist).

 Participants in all four FGDs made positive remarks regarding the framework and its criteria, notably their comprehensiveness. They explicitly mentioned the importance of separating individual and population perspectives regarding health benefits and harms, the range of feasibility considerations, and the broad perspective beyond mere health implications of an intervention. No general critical remarks about the framework were made in the FGDs.

###  Suggestions Towards Modifying the WHO-INTEGRATE Framework 

Table 2 provides an overview of suggestions for improvement derived from the KIIs or FGDs.

**Table 2:** Overview of Suggestions for Modifications of Framework, Criteria or Sub-criteria Based on FGDs and KIIs

**Criteria and Sub-criteria**	**Suggestions for Modifications of Framework, Criteria or Sub-criteria, Based on Statements in One or More FGDs and/or KIIs**			
	**Wording and Definition**	**Missing Aspects**	**Order and Grouping **	**Overlap, Redundancy and Delineation**
Balance of health benefits and harms		FGD		
Efficacy or effectiveness on health of individuals	FGD	FGD		
Efficacy or effectiveness on health of population				
Patients’/beneficiaries’ values in relation to health outcomes	KII, FGD	KII	KII	
Safety-risk-profile of intervention			FGD	
Broader positive or negative health-related impacts				
Human rights and socio-cultural acceptability		FGD	FGD	
Accordance with universal human rights standards	KII, FGD			
Socio-cultural acceptability to beneficiaries and those	KII, FGD	KII		KII, FGD
Socio-cultural acceptability of intervention to the public and other stakeholders	KII, FGD	FGD		
Impact on autonomy of concerned stakeholders				
Intrusiveness of intervention	FGD	FGD		
Equity, equality and non-discrimination	FGD	KII, FGD		FGD
Impact on health equality and/or health equity				
Distribution of benefits and harms of intervention				
Affordability of intervention	KII		FGD	KII
Accessibility of intervention				
Lack of a suitable alternative				
Societal implications	KII, FGD		FGD	
Social impact	KII, FGD			KII
Environmental impact	KII, FGD			
Financial and economic considerations				
Financial impact	KII	FGD		FGD
Impact on economy		FGD		
Ratio of costs and benefits		FGD		
Feasibility and health system considerations				
Legislation		KII		FGD
Leadership and governance	FGD	FGD		
Interaction with and impact on health system	FGD	FGD		KII
Need for, usage of and impact on health workforce and human resources		FGD		
Need for, usage of and impact on infrastructure	FGD	FGD		
Quality of evidence (meta-criterion)				
Suggestions regarding missing criteria		FGD		
Suggestions regarding the order of criteria				FGD

Abbreviations: FGD, focus group discussion; KII, key informant interview. An expanded version of this table is provided as a supplement. Supplementary file 4 details the suggested changes to the WHO-INTEGRATE framework based on KII and FGD, and Supplementary file 5 provides exemplary quotes based on the FGDs.

####  Wording and Definitions

 Participants in the KIIs and FGDs made several specific suggestions to expand upon and offer more guidance on selected criteria and sub-criteria; notably the criterion *Societal implications* was described as “fuzzy and vague” along with the sub-criteria *Accordance with human rights, Environmental implications *and* Intrusiveness of the intervention*.

*“These [criteria] [...] ‘impact on health system,’ ‘social impact,’ they are very vague” *(KII_WHO-Staff).

 Other suggestions included rewording “impact” to “implications,” distinguishing affordability more clearly from financial considerations, and clarifying the types of stakeholders that should be considered with respect to acceptability.

####  Missing Aspects

 Several interviewees stated explicitly that no criterion seemed to be missing in the framework; others suggested that the framework might not be sufficiently conducive for reflecting on underserved populations and vulnerable groups. They further recommended that a legal expert should assess whether supportive legal environments were sufficiently covered.

 FGD discussants noted several potentially missing aspects including intervention sustainability, reliability and quality of an intervention, and outcomes related to well-being, for instance:

 “*The benefits […] we define as […] professional[s] and [..] that adolescent[s] would define [..] is different: The pleasure of being together with a partner, physical contacts, enjoying beer and cigarette for them is special. I am not sure if [these] benefits [are] considered” *(FGD_Nepal).

 Furthermore, participants in the FGDs discussed whether political feasibility (eg, political and administrative facilitators and barriers) was sufficiently covered, in particular in regard to political feasibility on the local administrative and political level.

####  Order and Grouping of Criteria and Sub-criteria

 Several interviewees commented on the classification of *Patients’/beneficiaries’ values in relation to health outcomes* as a sub-criterion. As this is a (main) criterion in the current EtD framework,^27^ some interviewees were concerned that this aspect may not receive enough attention if only addressed as a sub-criterion.

*“I feel like [patients’/beneficiaries’ values in relation to health outcomes] is not really balance of benefits and harms. [...] So I wonder if maybe this can be part of the acceptability and values. Or something like that” *(KII_WHO-Staff).

 Discussants recommended a separation of *human rights and acceptability considerations* into two distinct criteria. Also, non-discrimination could be framed as a human rights consideration, rather than an aspect under *Equity and equality*. Furthermore, they suggested combining societal impact and health impact into one broad impact-oriented criterion.

####  Overlap, Redundancies and Delineation of Criteria and Sub-criteria

 Several interviewees commented on blurred boundaries between criteria and sub-criteria, eg, between the criterion *Health equity, equality and non-discrimination* and the sub-criterion *Social impact*, between the sub-criterion *Interaction with and impact on the health system* and the criterion *Financial and economic considerations, *as well as between acceptability considerations and the sub-criterion *Patients’/beneficiaries’ values in relation to health outcomes*.

*“I think it is just equity and non-discrimination and societal impact, there are some things that are overlapping. […] How would you really delineate?” *(KII_WHO-Staff).

 Participants in the FGDs whether the financial and economic as well as the resource considerations were adequately delineated in light of multiple payers on several geographical levels.

###  Relevance of Criteria and Sub-criteria Based on Focus Group Discussions Only

 Depending on the theme and topic of the FGD, different criteria dominated the discussions; nevertheless, references to all six criteria were identified in all four FGDs (Table 3, Supplementary file 6). For example, the FGD in Germany was dominated by the challenge to balance the intrusiveness of interventions and the resultant limitations inflicted on individual liberties with the potential health impacts and the available evidence. However, not all sub-criteria were discussed in every FGD. For example, implications for the (natural) environment were only explicitly discussed in the FGD in Uganda (focused on wastewater management). The most discussed themes included various (health) implications of the intervention, acceptability, accessibility, and autonomy and feasibility considerations. When asked, participants from all four FGD judged the framework to cover their reasoning well.

*“I think everything here needs to be kept. You’d rather furnish the decision-makers with more information than they need than less. And as far as I’m concerned, whatever’s in here would be really relevant” *(FGD_Uganda).

**Table 3:** Overview of Passages in the FGDs Containing a Reference to a Criterion or Sub-criteria Covered by the WHO-INTEGRATE Framework or Passages Mentioning a Criterion or Sub-criteria as Relevant for a Decision-Making Process

**Criteria and Sub-criteria**	**Brazil **	**Germany**	**Nepal**	**Uganda**
Balance of health benefits and harms	Yes	Yes	Yes	Yes
Efficacy or effectiveness on health of individuals	Yes			
Efficacy or effectiveness on health of population	Yes			Yes
Patients’/beneficiaries’ values in relation to health outcomes		Yes	Yes	
Safety-risk-profile of intervention	Yes			
Broader positive or negative health-related impacts			Yes	Yes
Human rights and socio-cultural acceptability	Yes	Yes	Yes	Yes
Accordance with universal human rights standards	Yes			Yes
Socio-cultural acceptability to beneficiaries and those	Yes		Yes	Yes
Socio-cultural acceptability of intervention to the public and other stakeholders	Yes	Yes	Yes	
Impact on autonomy of concerned stakeholders	Yes	Yes	Yes	Yes
Intrusiveness of intervention		Yes		Yes
Equity, equality and non-discrimination	Yes	Yes	Yes	Yes
Impact on health equality and/or health equity		Yes		Yes
Distribution of benefits and harms of intervention		Yes		Yes
Affordability of intervention	Yes		Yes	Yes
Accessibility of intervention	Yes	Yes	Yes	Yes
Lack of a suitable alternative		Yes		
Societal implications	Yes	Yes	Yes	Yes
Social impact			Yes	
Environmental impact				Yes
Financial and economic considerations	Yes	Yes	Yes	Yes
Financial impact	Yes		Yes	Yes
Impact on economy		Yes		Yes
Ratio of costs and benefits	Yes			Yes
Feasibility and health system considerations	Yes	Yes	Yes	Yes
Legislation	Yes	Yes	Yes	Yes
Leadership and governance		Yes		Yes
Interaction with and impact on health system	Yes			
Need for, usage of and impact on health workforce and human resources	Yes		Yes	
Need for, usage of and impact on infrastructure	Yes		Yes	
Quality of evidence (meta-criterion)	Yes	Yes	Yes	Yes

Abbreviations: FGD, focus group discussion; WHO, World Health Organization.

###  Implications for Using the WHO-INTEGRATE Framework in the WHO Guideline Development Process Based on Key Informant Interviews Only

 Several interviewees were concerned that the complexity and the additional workload associated with the WHO-INTEGRATE framework might be overwhelming for the guideline development process. This may lead to the process merely paying lip-service to criteria, such as *Societal implications*, and skipping over important domains. Budget constraints and limited time had to be considered when applying the framework.

*“I think that the guideline panels will find it [the expanded criteria and sub-criteria] more burdensome because to discuss all of these things will take longer. […] I think the panels get exhausted. They get tired and then they start skipping over, and they skip quite a lot” *(KII_Staff).

 In contrast, one interviewee stated that “cutting corners” to reduce the workload would diminish the value of the final product. This participant emphasized the need to raise the appropriate resources for a guideline to be “done right” and that using the framework as part of a well-coordinated process would not necessarily lead to a more expensive endeavor.

 Several participants stressed the need for additional guidance, including general guidance on how to apply the WHO-INTEGRATE framework and more specific guidance on how to select and interpret criteria and sub-criteria.

 Several interviewees further remarked that identifying appropriate evidence eg, for *Health system and feasibility considerations*, *Financial and Economic considerations, *or *Societal impact* might be challenging due to limited availability, low certainty and high context-dependency of evidence.

## Discussion

###  Discussion of Key Findings

 In this qualitative study, we received feedback on the WHO-INTEGRATE framework from WHO guideline developers as well as national public health and health policy decision-makers, and identified suggestions for modifications (Table 2, Supplementary file 5). Overall, the framework, its underlying conceptualization and its comprehensive nature, as well as the detailed criteria and sub-criteria were positively received. Some key informants voiced concerns regarding the implications of applying this framework with its very comprehensive set of criteria in everyday guideline development processes. A need for practical guidance was emphasized.

 All criteria and sub-criteria of the WHO-INTEGRATE framework were discussed or mentioned as relevant in at least one of the four FGDs (Table 3). Moreover, interviewees and participants in FGDs commented positively on the framework’s comprehensiveness. The developers of the WHO-INTEGRATE framework considered the few criteria highlighted to be missing by KII or FGD participants to be covered, pointing to a need to revise wording and provide clarification (see below). Since the FGDs and KIIs did not identify relevant gaps, this outcome aligns with the A4R framework’s^17^ condition of relevance, which depends on decision-makers using the framework properly. Our findings therefore suggest that the criteria included in the WHO-INTEGRATE framework can be considered both comprehensive and relevant for real-world public health decision-making.

 These findings are noteworthy in view of the heterogeneity of topics and settings in the FGDs and the diverse WHO guidelines selected for the KIIs (Table 3, Supplementary file 5). Likely, the overview of systematic reviews of real world decision criteria,^16,38^ undertaken to develop the WHO-INTEGRATE EtD framework played an important role: within the included systematic reviews,^9,10,44,45^ KIIs^9,46,47^ and FGDs^9,47-49^ were employed and the criteria used or suggested for use in these studies were similar to those discussed or mentioned in our KIIs and FGDs.^16,38^

 Beyond the aspects explicitly mentioned in KIIs and FGDs, the developers of the WHO-INTEGRATE framework (JMS, ER, IBS) noted some additional domains where a modification of the framework may be warranted. For example, while “individual well-being” was reported as missing in one FGD, the developers considered this aspect covered by the broad WHO concept of health, which was a key building block towards the WHO INTEGRATE framework. Similarly, the broad conceptualization of health systems^50^ (beyond healthcare systems) may not have been clear to all participants and may warrant changes in wording and definitions. Furthermore, in a future revision process the sub-criteria legislation and leadership and governance may need to be more fully described and the criteria relating to availability, accessibility or lack of a suitable alternative may need to be refined with reference to complex public health interventions (eg, labeling interventions).

 A concern voiced in the KIIs was the potential additional burden that the use of the WHO-INTEGRATE framework could impose on the guideline development process. This concern was not expressed in the FGDs. These different perceptions are also reflected in the literature: On the one hand, not adequately considering relevant criteria and the views of public health and health policy decision-makers as end-users was found to be a barrier to guideline adherence and implementation.^51,52^ On the other hand, the balancing act between rigorous methods and finite resources, notably limited time, repeatedly emerged as an obstacle in structured decision-making processes such as guideline development,^53,54^ and the necessity of pragmatic approaches is frequently emphasized.^53-55^ As highlighted in the publication of the WHO-INTEGRATE framework version 1.0,^16^ one solution to resolving the conflict between the comprehensiveness and granularity of the framework (both well-received) and the implications for guideline development (viewed with some concern) is to insist on a broad approach by considering all six criteria as well as the meta-criterion *quality of evidence* while allowing for much flexibility in terms of sub-criteria to be considered and evidence to be collected towards these (or not).^56^

 While EtD frameworks can support decision-makers in identifying relevant criteria for public health and health policy decisions, this does not supersede the need to address the value-laden nature of these decisions in other parts of the process.^16^ The substantive criteria put forward in the WHO-INTEGRATE framework need to be integrated with procedural considerations that address issues of fairness, participation, transparency and the right to appeal,^6,14,17,22^ eg, as suggested in what is referred to as evidence-informed deliberative processes.^6,14,57^ Major efforts should be made to achieve a balanced composition of the committee preparing for or making decisions, and to ensure representation of affected stakeholders. A balanced committee using an appropriate framework is poised to produce fair and reasonable decisions that are perceived as acceptable and legitimate – a point emphasized by interviewees and participants in the FGDs alike.

###  Strengths and Limitations

 This study followed a comprehensive and rigorous approach to capture the perspectives of those developing WHO guidelines and those potentially adapting and implementing WHO guidelines and/or developing national recommendations across four different countries and continents. Participants represented diverse roles and backgrounds, and two modes of obtaining insights (KIIs and FGDs) were pursued. While the development of other frameworks for structured decision-making^58-62^ mostly employed confirmatory approaches (eg, surveys to rate the importance of criteria^63^), our FGDs pursued a more open-ended approach stimulating participants to reflect deeply on issues of relevance for a given topic and to discuss the framework, its structure and criteria more freely.

 While the analysis was led by a researcher of German origin, data collection and analysis were conducted with local researchers to allow for an inter-cultural and interdisciplinary perspective. A thematic area and topic of relevance were proposed by local experts as the basis for the FGD in each country. Despite these strengths, the discussion represented a theoretical decision-making scenario, and criteria other than those discussed might arise in real-world decision-making. Two FGDs (Nepal and Brazil) were translated and it cannot be ruled out that nuances of the discussions were lost in translation, although efforts have been made to preserve the meaning in the process (eg, through contextualization of statements by local researchers and involvement of the local researchers in the analysis). A further limitation is that we did not systematically assess whether participants had financial or non-financial conflicts of interest in relation to the topic in all four FGDs, and analyzed how this may have affected their positioning in the discussion.

 Given the multiple dimensions of heterogeneity present in the FGDs (notably in terms of countries and topics), it is difficult to assess whether saturation was reached. Due to the differences in the nature of the topics, the discussions focused on different aspects and criteria (eg, the FGD in Nepal concerned with sexual and reproductive health did not address environmental implications, while this was an important consideration in the FGD in Uganda concerned with the management of untreated wastewater).^64^ Additional FGDs (among others in the WHO Western Pacific region) on an even broader set of topics might provide further considerations for advancing the framework. While we captured the perspective of a relatively small sample of users (four FGDs) and developers (nine KIIs), as discussed above, the WHO-INTEGRATE framework itself ^65^ builds on a much more comprehensive sample of similar real-world events.^38^

## Conclusion

 Our study suggests that the WHO-INTEGRATE framework can be a valuable resource for better-informed public health and health systems decisions. Reacting to the suggestions for improvement made by potential end-users, the developers are in the process of developing practical guidance for applying the WHO-INTEGRATE framework. Moreover, the applicability and added value of the framework will need to be tested in real-world guideline development and other decision-making processes, as planned with several upcoming WHO guidelines. The WHO-INTEGRATE framework was explicitly published as a living document. The findings presented here provide a valuable starting point to advance the framework towards a version 2.0.

## Acknowledgements

 The authors would like to thank all focus group participants and all key informants for their time and engagement. We are very grateful to Susan L. Norris, Anayda Portela, and Rebekah Thomas Bosco as well as other WHO staff for helping with the identification of suitable guidelines and key informants. Moreover, we would like to thank John Ssempebwa for facilitating the FGD in Uganda, and local assistants Monica Lima and Pedro Bossonario involved with supporting the authors AAM and CEMR in conducting the FGDs in Brazil. We are also grateful to the student assistants Sarah Thumbeck and Katja Krug for contributing to data analysis.

## Ethical issues

 Ethical approval was obtained from the Research Ethics Review Committees (RERC) of the WHO and the LMU Munich, as well as from local RERCs in Brazil, Nepal, and Uganda. All participants provided written and verbal consent prior to participation.

## Competing interests

 JMS and IBS: Developer of the WHO-INTEGRATE EtD framework. EAR: Developer of the WHO-INTEGRATE EtD framework, member of the GRADE Working Group. JMS reports grants from WHO Department of Maternal, Newborn, Child and Adolescent Health, personal fees from Bavarian Health and Food Safety Authority, other from Norwegian Agency for Development Cooperation (NORAD), during the conduct of the study.

## Authors’ contributions

 JMS and EAR designed and directed the project with contribution from IBS. JMS developed with interview guide for KIIs with input from EAR and IBS. JMS and EAR recruited the participants with the help from Susan L. Norris, Anayda Portela, Rebekah Thomas Bosco, and other WHO staff. JMS conducted the KIIs. KII-transcripts were analyzed by JMS with contribution from IBS, ST, and KK. DP, JO; KES, CEMdR, AAM, MF suggested topic and recruited participants for the FGDs as well as prepared and facilitated focus group discussions. DP, JO, KES, CEMR, AAM, MF revised and adapted the discussion guide drafted by JMS. JS supported this process in Uganda and ML and PB supported the facilitation of the FGDs in Brazil. JMS, DP, SES, CEMR, and AAM conducted the FGDs. DP, JO, KES, CEMR, AAM transcribed, translated and contextualized the FGD-audio records. JMS analyzed the FGD transcripts with contribution with contribution from IBS, ST, and KK, as well as input from all authors. JMS took the lead in writing the manuscript with input from all authors. All authors provided critical feedback and helped shape the research, analysis and manuscript.

## Funding

 The WHO-INTEGRATE EtD Framework Version 1.0 was developed with funding provided by the WHO Department of Maternal, Newborn, Child and Adolescent Health through grants received from the United States Agency for International Development and the Norwegian Agency for Development Cooperation. We also gratefully acknowledge that JMS’s position was funded by the Bavarian Health and Food Safety Authority, and that IBS’s input was funded by the Norwegian Agency for Development Cooperation (NORAD). The United States Agency for International Development, the Bavarian Health and Food Safety Authority and NORAD did not have any influence on the research process or content of this manuscript.

## Authors’ affiliations

^1^Institute for Medical Information Processing, Biometry, and Epidemiology – IBE, LMU Munich, Munich, Germany. ^2^Pettenkofer School of Public Health, Munich, Germany. ^3^Save the Children, Kathmandu, Nepal. ^4^The Water Institute, Department of Environmental Sciences and Engineering, The University of North Carolina at Chapel Hill, Chapel Hill, NC, USA. ^5^National Supplementary Health Agency, Ministry of Health, Brasília, Brazil. ^6^Department of Maternal-Infant Nursing and Public Health, College of Nursing, University of São Paulo, Ribeirão Preto, Brazil. ^7^Department of Disease Control and Environmental Health, Makerere University School of Public Health, Kampala, Uganda. ^8^Department of Global Health, Norwegian Institute of Public Health, Oslo, Norway. ^9^Bavarian Health and Food Safety Authority, Munich, Germany.

## Supplementary files


Supplementary file 1. Interview Guide for KIIs and FGDs.

Supplementary file 2. Details on KIIs and FGDs.

Supplementary file 3. Coding Frames Used to Code Transcripts of KIIs and FGDs.

Supplementary file 4. Suggested Changes to the WHO-INTEGRATE Framework Based on KIIs and FGDs.

Supplementary file 5. Suggested Changes to the WHO-INTEGRATE Framework Based on FGDs With Exemplary Quotes.

Supplementary file 6. Coverage of Decision Aspects Stated in the FGDs by Criteria and Sub-criteria of the WHO-INTEGRATE Framework.
