# Supplementary material for: Advancing the WHO-INTEGRATE Framework as a Tool for Evidence-Informed, Deliberative Decision-Making Processes: Exploring the Views of Developers and Users of WHO Guidelines
Source: Int J Health Policy Manag. 2020 Oct 27;11(5):629–41. doi: 10.34172/ijhpm.2020.193 (PMC9309924; doi:10.34172/ijhpm.2020.193)
Supplement: Supplementary file 1 — Interview Guide for KIIs and FGDs. [file ijhpm-11-629-s001.pdf]

Supplementary file 1. Interview Guide for KIISs and FGDs.

1. Key Informant Interview Guide - p. 1
2. FGD Guide – Brazil p. 4
3. FGD Guide – Germany p. 8
4. FGD Guide – Nepal p. 15
5. FGD Guide – Uganda p. 21

Key Informant Interview Guide

General Information

Staff:

	Name	Function
1)		Interviewer
2)		Research Assistant
3)		

Research question
• How do the participants evaluate the current and adapted EtR frameworks in terms of practical considerations?<br>• Which criteria and sub-criteria are missing from the adapted EtR framework? Which might not be needed?<br>• What implications does the adapted EtR framework have for evidence collection and guideline formulation?<br>• What would be sound methodological approaches to populate the criteria with evidence?

	Question / Activiy	Probing Questions	Aim
**1**	Please tell me about your experience with the guideline development process at the WHO	• Which EtR Framework was used in the guideline (Table 10.1; DECIDE EtR, other)?<br>How was your experience using this framework in the guideline development process?	Introduction into the topic<br><br>Get an insight on the experts knowledge and point of view on the issue;
**2**	In your opinion, what aspects do you consider as important to decision makers in their health care and health policy decision making, and therefore should be reflected in the guidelines?	• Which aspects do you regard as of particular importance to decision makers in relation to your recently completed/ongoing WHO guideline?<br>• Which normative criteria do you believe to be relevant for decision makers in regards to public health decisions?<br>• Which normative criteria do you believe to be relevant for decision makers in relation to your recently completed/ongoing WHO guideline in particular?	Capture criteria and categories which are perceived as relevant by the guideline developers.<br><br>Introduction into the topic
**3**	Which criteria and sub-criteria are missing from the current EtR framework?	• Are there any aspects which you believe to be relevant to the users of the WHO guidelines, but are not addressed in the current framework? Please explain	Assess how the participants evaluate the criteria in the current EtR frameworks
**4**	Which criteria and sub-criteria do you believe to be less relevant in the current EtR framework?	• Are there any aspects in the current framework which are less intensely covered in the EtR process? Why might this be the case?	
**5**	Please tell me about your experience with practicality of the EtR Framework used in your recently completed/ongoing WHO guideline (Table 10.1; DECIDE EtR, other)?	• How do you think is the understandability of the framework?<br>• How do you feel about the ease of use of the framework in the guideline development process?<br>• What is your opinion on the applicability of the framework at macro, meso, and micro decision making levels and their specific needs?<br>• What is your opinion on the order of the criteria and categories?	Assess how the participants evaluate the current EtR frameworks in terms of practical considerations

**6**	Provide the participant with the adapted EtR framework and invite the participant to read it thoroughly.<br><br>Please, tell me about your first impression of this adapted EtR framework	• Where do you see the main differences between the current and this adapted version of the EtR framework?<br>• How do you think is the understandability of this framework?<br>• How do you feel about the ease of use of the framework in the guideline development process?<br>• What is your opinion on the applicability of the framework at macro, meso, and micro decision making levels and their specific needs?	Assess how the participants evaluate the adapted EtR frameworks in terms of practical considerations
**7**	What is your opinion on the categories and criteria in the framework?	• What is your opinion on the order of the criteria and categories?<br>• Which criteria and sub-criteria are missing from the adapted EtR framework?<br>• Are any criteria not needed in the adapted EtR framework?<br>• Which criteria do you believe to be redundant?<br>• Which criteria do you believe to unnecessary for the users of WHO guidelines?	Assess how the participants evaluate the criteria in the adapted EtR frameworks
**8**	In contrast to the current practice, what implications will the adapted EtR framework have for evidence collection and guideline formulation?	• What implications will it have in regards to time and resource use and needs?<br>• What additional skills and capabilities of the technical teams will be needed in the adapted EtR Framework?	Assess the consequences of the adapted EtR framework on the guideline development process
**9**	What would be sound methodological approaches to populate the criteria with evidence?	• What are the methodological approaches used to populate the current WHO EtR Framework / the DECIDE EtR Framework with evidence?<br>• In regards to the additional normative criteria in the EtR framework, what methodological approaches seem to be most suitable to you?<br>• In regards to the additional technical criteria in the EtR framework, what methodological approaches seem to be most suitable to you?	Capture what the participants perceive as the most suitable way to populate the criteria in the adapted EtR framework with evidence

Guia preliminar para os Grupos Focais nos CCS (Country case studies)

Guia para Discussão de Grupo Focal (GF) no Brasil

Informações Gerais

Pessoal:

	Nome	Função
1	Carlos Rezende, MD PhD	Interviewer, Researcher
2	Aline Monroe, PhD	Interviewer, Researcher
3	Monica Lima	Note Taker and support
4	Pedro Bossonario	Note Taker and support

Data do GF: 29th, June 2018

Local do GF: Conference room in the building of the National Coordination for The National Tuberculosis Control Program (PNCT), MoH, Brasília, Brazil

Duração do GF: 150 min. with a 20 min. break

Idioma falado no GF: Portuguese

Instruções ao entrevistador
• Dê boas-vindas aos participantes do grupo focal.<br>• Apresente-se pelo nome e formação.<br>• Apresente o pesquisador (se não for a mesma pessoa que o entrevistador).<br>• Crie um ambiente acolhedor e descontraído.<br>• Agradeça aos participantes por dispensarem tempo para participar da pesquisa.<br>• Explique a finalidade do projeto de pesquisa "Fortalecendo o processo e métodos de recuperação, síntese e avaliação de evidências em intervenções complexas, multidisciplinares" e a finalidade do grupo de trabalho "Critérios da OMS para as evidências no processo de decisão"<br>• Explique o propósito do GF.<br>• Explique o que se pretende dizer com "programas e ações relacionadas a tuberculose e descentralização".<br>• Explique o que vai acontecer durante o GF.<br>• Estabeleça regras básicas para a discussão do GF (ou seja, apenas uma pessoa por vez, os participantes não devem interromper uns aos outros)<br>• Leia o formulário de consentimento informado e peça assinatura aos participantes do GF.<br>• Peça ao entrevistado se há alguma dúvida antes de iniciar o GF e responda a todas as perguntas.<br>• Explique por que a entrevista será gravada e peça permissão para tal. Comece a gravação.<br>• Comece a entrevista com a primeira pergunta.
Pergunta da pesquisa
• Como os participantes avaliam os modelos estruturados de "evidência para decisão" atuais e adaptados em termos de considerações práticas?<br>• Quais critérios e subcritérios estão faltando nos modelos estruturados adaptados de "evidência para decisão"? Quais não são necessários?<br>• Que implicações tem o modelo estruturado adaptado de "evidência para decisão" para a coleta de evidências e formulação de *guidelines*?<br>• O que seria considerado como abordagens metodológicas para preencher os critérios com evidência?
Tópico do Grupo Focal
• Programas de tuberculose e ações descentralizadas no Brasil

	Perguntas	Perguntas de sondagem	Objetivos
**Aquecimento & Introdução**			
**1**	Primeiro, eu gostaria que todos se apresentassem.<br><br>Vocês poderiam nos dizer seus nomes, profissão e de que forma você trabalhou em programas de tuberculose ou em descentralização no que diz respeito às medidas de saúde pública e cuidados de saúde?	• Como você definiria descentralização ou ações descentralizadas no que diz respeito à saúde pública e sistemas de saúde?	Apresentar os participantes uns aos outros<br>Introdução ao tópico<br>Obter insights sobre o conhecimento e a perspectiva do participante
**Decision-making criteria**			
**2**	Você acredita que os programas de tuberculose e a descentralização de suas ações no Brasil são importantes e por quê?	• Quais as principais razões para ser a favor da descentralização das ações dos programas de tuberculose?<br>• Quais as principais razões para ser contra a descentralização das ações dos programas de tuberculose?<br>• Quais aspectos éticos, sociais ou culturais você leva em conta quando apoia ou se opõe a descentralização das ações dos programas de tuberculose no Brasil?<br>• Quais argumentos técnicos ou financeiros você acredita ser relevante para apoiar ou se opor à descentralização dos programas de tuberculose?<br>• Que principais razões para apoiar ou opor-se a descentralização no sistema de saúde, a qual pode não ser relevante no diz respeito aos programas de tuberculose em particular?	Avaliar o que os participantes percebem como critérios relevantes para a tomada de decisão
**3**	Qual papel os guidelines da OMS desempenham no processo de tomada de decisão?	• Qual o papel que as recomendações dos guidelines da OMS exercem no processo de tomada de decisão?<br>• Quais informações você acredita serem úteis nos guidelines da OMS sobre a descentralização dos sistemas de saúde ou programas de tuberculose?<br>• Quais informações estão faltando nas diretrizes da WHO sobre programas de tuberculose e sobre a descentralização de suas ações no Brasil?<br>•	Avaliar como participantes avaliam as recomendações dos guidelines da OMS e os critérios utilizados nela
**4**	Além dos aspectos já mencionados, que critérios você acredita que precisam ser levados em conta quando uma	• Quais aspectos normativos que você acha que são relevantes?<br>• Quais aspectos técnicos ou financeiros que você acha que são	Avaliar o que os participantes percebem como critérios

	intervenção no sistema de saúde ou na saúde pública é realizada?	relevantes?	relevantes para a tomada de decisão
**Quadro adaptado de "evidências para decisão"**			
**6**	Forneça ao participante quadro adaptado de "evidência para decisão" e convide-o a lê-lo completamente.<br><br>Por favor, me diga sua primeira impressão deste quadro	• De que forma o quadro reflete seus pensamentos e a discussão geral sobre os programas de tuberculose e a descentralização de suas ações no Brasil?	Avaliar a primeira impressão dos participantes sobre o quadro adaptado de "evidência para decisão"
**7**	Qual a sua opinião sobre as categorias e critérios que aparecem no quadro?	• Qual a sua opinião sobre a ordem dos critérios e categorias?<br>• Quais critérios e subcritérios estão faltando do quadro adaptado de evidência para decisão?<br>• Todos os critérios não são necessários no quadro?<br>• Que critérios você acredita ser redundante?<br>• Que critérios acredita ser desnecessários para seu trabalho?	Analisar como os participantes avaliam os critérios constantes no quadro adaptado de "evidência para decisão"
**8**	Você acha que esse quadro é útil ao pensar sobre a introdução de programas de tuberculose, suas ações descentralizadas no Brasil e/ou descentralização dos sistemas de saúde em geral no Brasil?	• Você acha este quadro compreensível?<br>• Como você sente sobre a facilidade de uso do quadro quando pensa em introduzir intervenções de saúde pública?<br>• Qual a sua opinião sobre a aplicabilidade do quadro âmbito nacional, distrital ou do programa com suas necessidades específicas?	Analisar como participantes avaliam os quadros adaptados de "evidência para decisão" em termos de considerações práticas
**8**	Você acredita que um *guideline* da OMS que contempla os critérios do quadro pode ser mais útil para o seu processo de tomada de decisão? Por quê?	• Você acha que esses critérios são uma base útil para os *guidelines* desenvolvidos em seu país sobre os programas de tuberculose, suas ações descentralizadas e/ou sobre a descentralização dos sistemas de saúde em geral? Por quê?<br>• Você acha que esses critérios são uma base útil para os *guidelines* desenvolvidos em seu país no geral? Por quê?	Avaliar se os participantes percebem o quadro como útil
Instruções ao entrevistador			
• Agradeça novamente pelo tempo e disposição para realizar o GF.<br>• Pergunte se há alguma dúvida e responda todas as perguntas.<br>• Pare a gravação e se despeça.			

Annex II: Interview Guide for Focus Group Discussions for Country Case Study in Bavaria, Germany

Focus Group Discussion (FGD) Guide

General Information

Staff:

	Name	Function
1)	Jan M Stratil	Interviewer / Researcher
2)		
3)		

Date of FGD: 07. Juni 2018

Location of FGD: Munich, Germany (Oberschleißheim)

Duration of FGD 90 Minutes

Language spoken: German

Interviewer Instructions
• Welcome the participants of the focus group<br>• Introduce yourself by name and function.<br>• Introduce researcher (if not the same person as the interviewer)<br>• Create a warm and easy-going atmosphere.<br>• Thank the participants for taking the time to take part in this research.<br>• Explain the purpose of the overall research project "Strengthening the process and methods for retrieval, synthesis and assessment of evidence on complex, multidisciplinary interventions" and the purpose of the Working Group on the "WHO criteria for the evidence to recommendations process".<br>• Explain the purpose and the topic of the FGD.<br>• Explain what will happen during the FGD.<br>• Set out ground rules for the focus group discussion (i.e. only one person speaks at the time, participants should not interrupt each other)<br>• Go through the informed consent form and have the FGD-participants sign the statement.<br>• Ask the interviewee if there are any questions before starting the FGD and answer all questions.<br>• Explain why the interview will be tape recorded and ask for permission. Start the tape.<br>• Start the interview with the first question.
Research question
● Do the WHO-INTEGRATE framework and its decision criteria reflect the needs of national and sub-national decision makers on health as potential users of WHO-guidelines?<br>● Which criteria do national and sub-national decision makers on health as potential users of WHO guidelines generally consider important in health care and health policy decision making?<br>● How do the participants evaluate the criteria in the WHO-INTEGRATE EtD framework?<br>● How do the participants evaluate the WHO-INTEGRATE EtD framework in terms of practical considerations such as usability and understandability?

Topic of the FGD			
● Interventions to reduce a potential negative health impact of an increased market share of Isoglucose in Germany following EU-Regulation.<br>● Intervention to be discussed: (1)			
	**Questions**	**Probing Questions**	**Aims**
Warm-up & Introduction			
**1**	First, I would like everyone to introduce themselves.<br>*Ich würde gerne mit einer Vorstellungsrunde beginnen*<br><br>Can you tell us your name, Institution and in what way you or your organization is involved or affected by the deregulation of Isoglucose limits?<br>*Können Sie sich bitte kurz Vorstellen mit Name, Institution die Sie hier vertreten und in welcher in aller Kürze darstellen in wie Ihre Institution von der Deregulation der Obergrenze für Isoglukose betroffen ist?*		Introduce participants to each other<br>Introduction to the topic<br>Obtain insights regarding knowledge and perspective of participant
**Decision-making criteria**			
**2**	Do you believe **countermeasures in general** to react to an increased market share of Isoglucose are important and why?<br>*Halten Sie es grundsätzlich für nötig Maßnahmen in Bezug auf einen höheren Marktanteil von Isoglukose zu treffen? Und Weshalb?*	• What do you believe are the main reasons to support the introduction of counter-measures in general?<br>*Was meinen Sie sind wichtige Gründe dafür Maßnahmen in Reaktion auf einen gesteigerten Marktanteil von Isoglukose zu unterstützen?*<br>• What do you believe are the main reasons to oppose the introduction of counter-measures in general?<br>*Was meinen Sie sind wichtige Gründe dafür Maßnahmen in Reaktion auf einen gesteigerten Marktanteil von Isoglukose abzulehnen?*<br>• What normative aspects do you taken into account when you support or oppose the introduction of counter-measures in general?<br>*Welche Rolle spielen normativ-ethische Gründe bei der Entscheidung für oder gegen Interventionsmaßnahmen?*<br>• Which technical or financial arguments do you believe to be of relevance when opposing or supporting the introduction of counter-measures in general?<br>*Welche Rolle spielen technische oder finanzielle Gründe bei der Entscheidung für oder gegen Interventionsmaßnahmen?*	Assess what participants perceive as relevant criteria for decision making

	Do you believe **labeling** of the Isoglucose content on food and beverages would be a suitable countermeasure?<br>*Halten Sie eine **Kennzeichnungspflicht** von Isoglukose auf Lebensmitteln und Getränken für eine geeignete Gegenmaßnahme?*	• What do you believe are the main reasons to support the introduction of **Isoglucose food and beverage labeling**?<br>*Was meinen Sie sind wichtige Gründe dafür eine Kennzeichnungspflicht von Isoglukose auf Lebensmitteln und Getränken zu unterstützen?*<br>• What do you believe are the main reasons to oppose the introduction of **Isoglucose food and beverage labeling**?<br>*Was meinen Sie sind wichtige Gründe dafür eine **Kennzeichnungspflicht von Isoglukose auf Lebensmitteln und Getränken abzulehnen**?*<br>• What normative aspects do you taken into account when you support or oppose the introduction of **Isoglucose food and beverage labeling**?<br>*Welche Rolle spielen normativ-ethische Gründe bei der Entscheidung für oder gegen die Einführung von **Kennzeichnungspflichten von Isoglukose auf Lebensmitteln und Getränken**?*<br>• Which technical or financial arguments do you believe to be of relevance when opposing or supporting the introduction of **Isoglucose food and beverage labeling**?<br>*Welche Rolle spielen technische oder finanzielle Gründe bei der Entscheidung für oder gegen die Einführung von **Kennzeichnungspflichten von Isoglukose auf Lebensmitteln und Getränken**?*<br>•	
	Do you believe a continuation of the Isoglucose-Quota (e.g. on national level) would be a suitable countermeasure?<br>*Halten Sie eine Fortführung der Quotenregelung bezüglich Isoglukose (z.B. auf Nationalstaatlicher Ebene) für eine geeignete Gegenmaßnahme?*	• What do you believe are the main reasons to support **continuation of the Isoglucose-Quota** (e.g. on national level)?<br>*Was meinen Sie sind wichtige Gründe dafür die **Fortführung der Quotenregelun**g bezüglich Isoglukose zu unterstützen?*<br>• What do you believe are the main reasons to oppose the **continuation of the Isoglucose-Quota**?<br>*Was meinen Sie sind wichtige Gründe dafür die **Fortführung der Quotenregelung** bezüglich Isoglukose abzulehnen?*<br>• What normative aspects do you taken into account when you support or oppose the **continuation of an Isoglucose-Quota**?<br>*Welche Rolle spielen normativ-ethische Gründe bei der Entscheidung für oder gegen die die **Fortführung der Quotenregelung** bezüglich Isoglukose?*<br>• Which technical or financial arguments do you believe to be of relevance when opposing or supporting the **continuation of an Isoglucose-Quota**?<br>*Welche Rolle spielen technische oder finanzielle Gründe bei der Entscheidung für oder gegen die die **Fortführung der Quotenregelung** bezüglich Isoglukose?*	

	Do you believe a selective **taxation** of the Isoglucose content on food and beverages would be a suitable countermeasure?<br>*Halten Sie eine **selektive Besteuerung** von Isoglukose in Lebensmitteln und Getränken für eine geeignete Gegenmaßnahme?*	• What do you believe are the main reasons to support the introduction of **Isoglucose food and beverage labeling**?<br>*Was meinen Sie sind wichtige Gründe dafür eine **selektive Besteuerung** von Isoglukose in Lebensmitteln und Getränken zu unterstützen?*<br>• What do you believe are the main reasons to oppose the introduction of **Isoglucose food and beverage labeling**?<br>*Was meinen Sie sind wichtige Gründe dafür eine **selektive Besteuerung** von Isoglukose in Lebensmitteln und Getränken abzulehnen?*<br>• What normative aspects do you taken into account when you support or oppose the introduction of **Isoglucose food and beverage labeling**?<br>*Welche Rolle spielen normativ-ethische Gründe bei der Entscheidung für oder gegen die Einführung einer **selektiven Besteuerung** von Isoglukose in Lebensmitteln und Getränken?*<br>• Which technical or financial arguments do you believe to be of relevance when opposing or supporting the introduction of **Isoglucose food and beverage labeling**?<br>*Welche Rolle spielen technische oder finanzielle Gründe bei der Entscheidung für oder gegen die Einführung einer **selektiven Besteuerung** von Isoglukose in Lebensmitteln und Getränken?*	
**3**	What roles do WHO-guidelines play in the decision-making process within your institution?<br>*Welche Rolle spielen WHO Leitlinien in so einem Entscheidungsprozess innerhalb Ihrer Institution?*		Assess how participants evaluate the WHO guideline recommendations and the criteria used in it?
**4**	Beside the aspects already mentioned, what criteria do you believe have to be taken into account when reflecting on whether a public health or health system intervention should be introduced?<br>*Neben den schon genannten Aspekten, welche weiteren Aspekte sollten in Betracht gezogen werden wenn über Gesundheitspolitische oder sonstige Maßnahmen zur Förderung der öffentlichen Gesundheit diskutiert wird?*	• What normative aspects do you think are of relevance?<br>*Welche weiteren normativen Aspekte scheinen Ihnen in Bezug auf Gesundheitspolitische oder sonstige Maßnahmen zur Förderung der öffentlichen Gesundheit als relevant?*<br>• What technical or financial aspects do you believe are of relevance?<br>*Welche weiteren technischen oder finanziellen Aspekte scheinen Ihnen in Bezug auf Gesundheitspolitische oder sonstige Maßnahmen zur Förderung der öffentlichen Gesundheit als relevant?*	Assess what participants perceive as relevant criteria for decision making

Adapted EtD framework			
**5**	Provide the participant with the adapted EtD framework and invite the participant to read it thoroughly.<br><br>Please, tell me about your first impression of this framework<br>*Was kommt Ihnen als erstes in den Sinn, wenn Sie dieses Entscheidungsframework sehen?*	• In what way does the framework reflect your thoughts and the general discussion about the interventions on Isoglucose?<br>Auf welche Weise sind Ihre Gedanken und unsere bisherige Diskussion zu Interventionen in Bezug auf Isoglukose in dem Framework abgebildet?	Assess the first impression of the participants on the revised EtD framework?
**6**	What is your opinion on the categories and criteria in the framework?<br>*Was ist Ihre Meinung zu den Kategorien und Kriterien in dem Framwork?*	• Which criteria and sub-criteria are missing from the WHO-INTEGRATE EtD framework?<br>*Welche Kriterien oder Subkriterien fehlen Ihnen in dem WHO-INTEGRATE EtD Framework?*<br>• Are any criteria not needed in framework?<br>*Erachten Sie irgendwelche Kriterien in dem Framework als überflüssig? Wenn ja: welche?*<br>• Which criteria do you believe to be redundant?<br>*Erachten Sie irgendwelche Kriterien in dem Framework als redundant? Wenn ja: welche?*<br>• Which criteria do you believe to be unnecessary for your work and why?<br>*Welche Kriterien erachten Sie als überflüssig und weshalb?*<br>• What is your opinion on the order of the criteria and categories?<br>*Wie ist Ihre Meinung zu der Reihenfolge der Kriterien und Kategorien?*	Assess how the participants evaluate the criteria in the adapted EtD frameworks
**7**	Do you think this framework is useful when thinking about the introduction of interventions to counter a potential negative population health impact of Isoglucose? Why or why not?<br>*Halten Sie das Framework für nützlich, wenn Sie an die Reflexion über Interventionen zur Reaktion auf einen möglicherweise erhöhten Marktanteil von Isoglukose denken? Warum bzw. warum nicht?*	• How do you think is the understandability of this framework?<br>*Wie schätzen Sie die Verständlichkeit des Frameworks ein?*<br>• How do you feel about the ease of use of the framework when thinking about introducing public health interventions?<br>*Wie erachten Sie die Anwendungsfreundlichkeit des Frameworks?*<br>• What is your opinion on the applicability of the framework at national, district or program level their specific needs?<br>*Was ist Ihre Meinung zu der Anwendbarkeit des Frameworks auf nationaler, regionaler oder kommunaler Entscheidungsfindung zu Gesundheitsinterventionen?*	Assess how participants evaluate the adapted EtD frameworks in terms of practical considerations

**8**	Do you believe, a WHO guideline which takes the criteria of the framework into account may be useful for your decision-making process? Why?<br>*Schätzen Sie eine WHO Leitlinie, welche die Kriterien im Frameworks berücksichtigt für nützlich in Ihrer Entscheidungsfindung? Warum bzw. warum nicht?*	• Do you think these criteria are a useful basis for guidelines developed in Germany on isoglucose-issues? why?<br>*Halten Sie die im Framework enthaltenen Kriterien für eine nützliche Basis bei der Entwicklung von Leitlinien zu Isoglukose in einem deutschen Kontext? Warum bzw. warum nicht?*<br>• Do you think these criteria are a useful basis for guidelines developed in Germany in general? Why?<br>*Halten Sie die im Framework enthaltenen Kriterien für eine nützliche Basis bei der Entwicklung von Leitlinien im Allgemeinen in einem deutschen Kontext? Warum bzw. warum nicht?*	Assess whether the participants perceive the framework as useful

Interviewer Instructions

- Thank again for the time and willingness for the FGD.
- Ask if there are any questions left and answer all questions.
- Stop the tape and say goodbye.

Focus Group Discussion (FGD) Guide in Nepal

केन्द्रिकृत समुह छलफल निर्देशिका

General Information<br>सामान्य जानकारी

Staff:

	Name<br>नाम	Function<br>कार्य
1)	Deepak Paudel<br>दिपक पौडेल	Interviewer<br>छलफल सहजकर्ता:
2)	Jan Stratil	Interviewer and/or Note Taker<br>छलफल टिप्पणीकर्ता:
3)		Researcher<br>छलफल टिपोटकर्ता:

Date of FGD: ___ of August, 2017

छलफलको मिति:

Location of FGD:

छलफलको स्थान:

Duration of FGD

छलफलको अवधि:

Language spoken: Nepali

भाषा: नेपाली

Interviewer instructions<br>निर्देशन
• Welcome the participants in the focus group<br>सहभागीलाई स्वागत गर्ने<br>• Introduce yourself by name and function.<br>परिचय दिने र भुमिका बताउने<br>• Introduce researcher (if not the same person as the interviewer)<br>अनुसन्धानकर्ता को परिचय<br>• Create a warm and easy-going atmosphere.<br>सौहार्दपूर्व वातावरण तयार गर्ने<br>• Thank the participants for taking the time to take part in this research.<br>सहभागीहरुलाई उनिहरुको समयको लागि धन्यवाद ज्ञापन गर्ने<br>• Explain the purpose of the research project “Strengthening the process and methods for retrieval, synthesis and assessment of evidence on complex, multidisciplinary interventions” and the purpose of the Working Group on the “WHO criteria for the evidence to recommendations process”.<br>अध्ययनको उद्धेश्य बताउने<br>• Explain the purpose and the topic of the FGD.<br>छलफलको उद्धेश्य र विषयवस्तु बताउने<br>• Explain what will happen during the FGD.<br>छलफलको प्रकृया बताउने<br>• Lay out ground rules for the FGD (i.e. only one person speaks at the time, participants should not interrupt each other)<br>छलफलका नियमहरु ब्ताउने<br>• Go through the informed consent form and have the FGD participants sign the statement.<br>स्विकृती फारमको बारेमा बताउने र फारम भर्ने<br>• Ask the participants if there are any questions before starting the FGD and answer all questions.

सहभागीको केहि जिज्ञासा भए सोध्ने र प्रष्ट पार्ने

- Explain why the FGD will be tape recorded and ask for permission. Start the tape.
  छलफलको रेकर्डिङ्ग को उद्वेश्य बताउने र स्विकृती लिने । रेकर्डिङ्ग शुरु गर्ने
- Start the FGD with the first question.
  छलफल शुरु गर्ने ।

Research questions
अनुसन्धानका प्रश्नहरु

- Which criteria do the participants perceive as relevant in regards to decision making on [**TOPIC**]
  प्रस्तुत विषयवस्तु मा आवश्यक निर्णय गर्न के कुरा सान्दर्भिक छ?
- How do the participants evaluate the current and adapted EtD frameworks in terms of practical considerations?
  व्यवहारीक रुपमा हालको र नयाँ सुचनामा आधारित निर्णय गर्ने रुपरेखा ले के महत्व राख्दछ?
- Which criteria and sub-criteria are missing from the adapted EtD framework? Which might not be needed?
  हालको रुपरेखामा के छुटेको छ र के आवश्यक पर्दैन?

Topic of the FGD
प्रस्तुत विषयवस्तु

Information, counselling and services for reproductive health including contraception in Nepal, as part of the WHO The Global Strategy for Women's and Children's Health 2030
नेपालमा किशोरावस्थाका लागि गर्भनिरोधक सहित प्रजनन स्वास्थ्यको लागि जानकारी, परामर्श तथा सेवा, विश्व स्वास्थ्य संघको एक भागका रुपमा महिला तथा वाल स्वास्थ्यका लागि विश्व रणनीति २०३० ।

	**Questions**<br>प्रश्नहरु	**Probing Questions**<br>थप जानकारी	**Aims**<br>उद्वेश्य
**Warm-up and introduction**<br>सहजिकरण र विषय प्रवेश			

**1**	First, I would like everyone to introduce themselves.<br>अब हामीहरु परिचय वाट शुरु गरौं ।<br><br>Can you tell us your name, occupation and in what way you have worked on reproductive health?<br>तपाई आफ्नो नाम, पेशा, र प्रजनन स्वास्थ्य सँगको संलग्नता वताईदिनु होस् न ।		Introduce participants to each other<br>Obtain insights regarding knowledge and perspective of participant<br>सहभागीहरुलाई परिचित गराउनुहोस् र उहाँहरुको जानकारी र दृष्टिकोण को अन्तरदृष्टि खोज गर्नुहोस् ।
**Decision-making criteria**<br>निर्णय प्रकृया			
**2**	Do you believe interventions focusing on information, counselling and services for reproductive health including contraception is important in Nepal, and why?<br>गर्भनिरोधक र प्रजनन स्वास्थ्यका लागि जानकारी, सल्लाह र सेवाका सन्दर्भमा केन्द्रिकृत रहिमा नेपालको सन्दर्भमा के कुराले महत्व राख्दछ र किन?	• What do you believe are the main reasons to support the introduction of guidelines or other general recommendations for interventions focusing on information, counselling and services for reproductive health including contraception?<br>प्रजनन स्वास्थ्य र परिवार नियोजन विषयमा आवश्यक निर्देशिका तथा अन्य सुझावहरु दिनुको कारणहरु के हो जस्तो लाग्दछ?<br>• What do you believe are the main reasons to oppose the introduction of specific interventions focusing on information, counselling and services for reproductive health including contraception ?<br>गर्भनिरोधक र प्रजनन स्वास्थ्यका लागि जानकारी, सल्लाह र सेवाका सन्दर्भमा केन्द्रिकृत रहि उक्त निर्देशिकाहरु दिनुको विरोध किन हुन्छ जस्तो लाग्दछ?<br>• Which technical or feasibility arguments do you believe to be of relevance when opposing or supporting the introduction of specific interventions focusing on information, counselling and services for reproductive health including contraception ?<br>गर्भनिरोधक सहित प्रजनन स्वास्थ्यका लागि जानकारी, सल्लाह र सेवाका सन्दर्भमा केन्द्रिकृत रहि यस्ता निर्देशिकाहरु	Assess what participants perceive as relevant criteria for decision making<br>सहभागीहरुले के कुरालाई निर्णय प्रकृयामा उपयोगी महशुस गर्दछन् भनि लेखाजोखा गर्नुहोस् ।

		जारी गर्दा विरोध र समर्थन गर्दा के कस्ता प्राविधिक र सम्भाव्यताको आधारमा छलफल हुन्छ जस्तो लाग्दछ?<br>• What economic or financial considerations do you believe to be of relevance when opposing or supporting the introduction of specific interventions focusing on information, counselling and services for reproductive health including contraception ?<br>गर्भनिरोधक सहित प्रजनन स्वास्थ्यका लागि जानकारी, सल्लाह र सेवाका सन्दर्भमा केन्द्रिकृत रहि यस्ता निर्देशिकाहरु जारी गर्दा विरोध र समर्थन गर्दा के कस्ता आर्थिक र वित्तिय कारणहरुको आधारमा छलफल हुन्छ जस्तो लाग्छ?<br>• Which social and cultual aspects do you take into account when you support or oppose the introduction of specific interventions focusing on information, counselling and services for reproductive health including contraception<br>गर्भनिरोधक सहित प्रजनन स्वास्थ्यका लागि जानकारी, सल्लाह र सेवाका सन्दर्भमा केन्द्रिकृत रहि यस्ता निर्देशिकाहरु जारी गर्दा, विरोध र समर्थन गर्दा के कस्ता सामाजिक र सांस्कृतिक कारणहरुको आधारमा छलफल हुन्छ जस्तो लाग्छ?<br>• What ethical arguments are reflected when considering the support or the opposition of the introduction of specific interventions focusing on information, counselling and services for reproductive health including contraception<br>गर्भनिरोधक सहित प्रजनन स्वास्थ्यका लागि जानकारी, सल्लाह र सेवाका सन्दर्भमा केन्द्रिकृत रहि यस्ता निर्देशिकाहरु जारी गर्दा विरोध र समर्थन गर्दा के कस्ता नैतिक पक्षहरुको आधारमा छलफल हुन्छ जस्तो लाग्दछ?<br>• What role do the results from scientific research play in the considerations on the support or the opposition of the introduction of specific interventions focusing on information, counselling and services for reproductive health including contraception?	

		गर्भनिरोधक सहित प्रजनन स्वास्थ्यका लागि जानकारी, सल्लाह र सेवाका सन्दर्भमा केन्द्रिकृत रहि वैज्ञानिक अनुसन्धानहरुको नतिजाले यस्ता निर्देशिका जारी गर्दा कस्तो भूमिका खेल्दछन जस्तो लाग्छ?	
**3**	What role do WHO guidelines play in national decision-making processes on maternal and child health?<br>प्रस्तुत विषयमा विश्व स्वास्थ्य संघको निर्देशिका ले राष्ट्रिय स्तरमा निर्णय गर्न कस्तो भूमिका खेल्दछ?	• What role do the recommendations in WHO Guidelines play in national decision-making processes in the area of maternal and child health, especially adolescents' health?<br>विश्व स्वास्थ्य संघ द्वारा जारी यस्ता निर्देशिकाहरुमा सिफारिस गरिएका कुराहरुले राष्ट्रिय स्तरमा गर्ने निर्णयमा कत्तिको/कस्तो भूमिका खेल्दछन्?	Assess how participants evaluate the usefulness of WHO guideline recommendations<br>सहभागीहरुले विश्व स्वास्थ्य संघको निर्देशिका को उपयोगिता को लेखाजोखा गर्नुहोस ।
**Adapted EtD framework**<br>नयाँ सूचनामा आधारित निर्णय गर्ने रुपरेखा			
**6**	Provide the participants with the adapted EtD framework and invite them to read it thoroughly.<br>नया सुचनामा आधारित निर्णय गर्ने रुपरेखा प्रस्तुत गर्ने र सहभागिहरुलाई अध्ययन गर्न समय दिने ।<br>Please, tell me about your first impression of this framework.<br>तपाईलाई नयाँ रुपरेखा कस्तो लाग्यो?	• In what way does the framework reflect your thoughts and the general discussion about interventions focusing on information, counselling and services for reproductive health including contraception ?<br>गर्भनिरोधक सहित प्रजनन स्वास्थ्यका लागि जानकारी, सल्लाह र सेवाका सन्दर्भमा केन्द्रिकृत रहि निर्देशिकामा जारी सिफारिसहरु तपाईको विचार सँग कत्तिको मेल खायो?	Assess the first impression of the participants of the revised EtD framework<br>सहभागीहरुको पहिलो दृष्टिकोण परिमार्जित रुपरेखामा कस्तो छ भनि लेखाजोखा गर्नुहोस ।
	What is your opinion about the overall structure of the framework?<br>यस रुपरेखामा आवश्यक विषयवस्तु कसरी समेटिएको पाउनु भयो?	• How do you rate the relevance of the health benefits aspects?<br>स्वास्थ्यको लाभको हिसावले यो निर्देशिका कत्तिको उपयुक्त लाग्यो?<br>• To what extent are the acceptability aspects of relevance?<br>स्विकार्यताको हिसावले यो निर्देशिका कत्तिको उपयुक्त लाग्यो?<br>• To what extent are the equity aspects of relevance?<br>समावेशीताको हिसावले यो निर्देशिका कत्तिको उपयुक्त लाग्यो?	Assess how the participants evaluate the overall structure of the adapted EtD frameworks<br>उक्त रुपरेखाको संरचना सहभागीलाई कस्तो लाग्यो भनि लेखाजोखा गर्नुहोस ।

		• How do you rate the relevance of the long-term social, economic and environmental benefits aspects?<br>दिर्घकालिन सामाजिक, आर्थिक र वातावरणिय हिसावले यो निर्देशिका कत्तिको उपयुक्त लाग्यो?<br>• To what extent are the utility aspects of relevance?<br>उपयोगिताको हिसावले यो निर्देशिका कत्तिको उपयुक्त लाग्यो?<br>• To what extent are the feasibility aspects of relevance?<br>संम्भाव्यताको हिसावले यो निर्देशिका कत्तिको उपयुक्त लाग्यो?	
**7**	What is your opinion about the criteria and sub-criteria in the framework?<br>यस रुपरेखामा समेटिएका बुँदा र उपबुँदाहरु कस्तो लाग्यो?	• Are any criteria or sub-criteria missing from the adapted EtD framework? If so, which ones?<br>यस रुपरेखामा केहि मापदण्ड वा उप मापदण्ड छुटेका छन्? छन् भने के के ?<br>• Are any criteria or sub-criteria not needed in the framework? If so, which ones?<br>यस रुपरेखामा भएका कुनै मापदण्ड वा उप मापदण्ड आवश्यक छैनन्? छैनन् भने के के?	Assess how the participants evaluate the criteria in the adapted EtD frameworks<br>सहभागीहरुलाई नयाँ रुपरेखाको मापदण्ड कस्तो लाग्यो भनि लेखाजोखा गर्नुहोस ।
**8**	Do you think this framework is useful when thinking about the introduction of specific interventions focusing on information, counselling and services for reproductive health including contraception ?<br>गर्भनिरोधक सहित प्रजनन स्वास्थ्यका लागि जानकारी, सल्लाह र सेवाका सन्दर्भमा तपाईलाई यस रुपरेखाले निर्देशिकाको प्रस्तुतिमा कति उपयोगी भएको महशुस गर्नु भयो?	• How do you think is the understandability of this framework?<br>यस रुपरेखामा दिईएका विषयवस्तु कत्तिको बुझ्न सकिने छ?<br>• How do you feel about the ease of use of the framework when thinking about introducing public health interventions?<br>यस रुपरेखामा दिईएका विषयवस्तु कत्तिको सहज लाग्छ (जनस्वास्थ्यको कार्यक्रमको सन्दर्भमा)<br>• What is your opinion on the applicability of the framework at national, district or program level (your specific needs)?<br>यस रुपरेखाको उपादेयता राष्ट्रिय, जिल्ला र कार्यक्रमको तहमा कत्तिको उपयोगी छ? कतिको तपाईहरुको आवश्यकता अनुरुपको छ?	Assess how participants evaluate the adapted EtD frameworks in terms of practical considerations<br>व्यवहारिक दृष्टिकोणबाट सहभागीहरुलाई रुपरेखा कस्तो लाग्यो भनि लेखाजोखा गर्नुहोस ।

**8**	Do you believe a WHO guideline taking the criteria of the framework into account would be useful for your decision-making process? Why?<br>के तपाईलाई विश्व स्वास्थ्य संघको निर्देशिका र यसका वुँदाहरुले आवश्यक निर्णय लिने कार्यमा उपयोग हुन्छ जस्तो लाग्छ? किन?	• Do you think these criteria are a useful basis for guidelines adapted or developed in Nepal on [TOPIC]? Why?<br>के तपाईलाई यी निर्देशिकाहरु नेपालको रणनीति तयार गर्न उपयोगी ठान्नुभयो? किन?<br>• Do you think these criteria are a useful basis for other public health guidelines adapted or developed in Nepal? Why?<br>के यी मापदण्डहरु अरु जनस्वास्थ्य सम्बन्धि निर्देशिका तयार गर्न उपयोगी ठान्नुभयो? किन?	Assess whether the participants perceive the framework as useful/not useful<br>उक्त रुपरेखा सहभागीलाई उपयोगी लागे नलागेको लेखाजोखा गर्नुहोस ।

Interviewer Instructions
अनुसन्धानकर्तालाई निर्देशन

- Thank again for the time and willingness to participate in the FGD.
  सहभागीको समयको लागि पूनः धन्यवाद दिने ।
- Ask if there are any questions left and answer all questions.
  सहभागीको केहि जिज्ञाशा भए सोध्ने र प्रष्ट पार्ने ।
- Stop the tape and say goodbye.
  रेकर्डिङ्ग वन्द गर्ने र विदा हुने ।

Focus Group Discussion (FGD) Guide in Uganda

General Information

Staff:

	Name	Function
1)	Jimmy Osuret	Interviewer and/or Note Taker
2)	Karen Setty	Interviewer and/or Note Taker
3)	John Ssempebwa	Researcher

Date of FGD: August 4, 2017

Location of FGD: WHO offices (room to be confirmed)

Duration of FGD 10:00 AM to 12:00 PM (2 hours)

Language spoken: English

Interviewer instructions
• Welcome the participants in the focus group<br>• Introduce yourself by name and function.<br>• Introduce researcher (if not the same person as the interviewer)<br>• Create a warm and easy-going atmosphere.<br>• Thank the participants for taking the time to take part in this research.<br>• Explain the purpose of the research project "Strengthening the process and methods for retrieval, synthesis and assessment of evidence on complex, multidisciplinary interventions" and the purpose of the Working Group on the "WHO criteria for the evidence to recommendations process".<br>• Explain the purpose and the topic of the FGD.<br>• Explain what will happen during the FGD.<br>• Lay out ground rules for the FGD (i.e. only one person speaks at the time, participants should not interrupt each other)<br>• Go through the informed consent form and have the FGD participants sign the statement.<br>• Ask the participants if there are any questions before starting the FGD and answer all questions.<br>• Explain why the FGD will be tape recorded and ask for permission. Start the tape. Ask participants to speak one at a time and loud enough to be heard by recorder. Also put cell phones on vibrate to avoid noisy interruptions.<br>• Start the FGD with the first question.
Research questions
● How do the participants evaluate the current and adapted EtD frameworks in terms of practical considerations?<br>● Which criteria and sub-criteria are missing from the adapted EtD framework? Which might not be needed?
Topic of the FGD
● Management of untreated wastewater, including sewage from septic tanks and fecal sludge from pit latrines will be an important challenge facing Uganda and other developing nations over the next several years. Sustainable Development Goal 6 target 6.3 seeks to, "By 2030, improve water quality by reducing pollution, eliminating dumping and minimizing release of hazardous chemicals and materials, halving the proportion of untreated wastewater and substantially increasing recycling and safe reuse globally." WHO is in the process of developing guidelines on sanitation, and countries will be tasked to adapt and implement these at national and sub-national levels.

	Questions	Probing Questions	Aims
**Warm-up and introduction**			
**1**	First, I would like everyone to introduce themselves.<br><br>Can you tell us your name, occupation and in what way you have worked on managing wastewater, septage, or sludge?		Introduce participants to each other<br>Obtain insights regarding knowledge and perspective of participant
**Decision-making criteria**			
**2**	Do you believe managing wastewater, septage, or sludge is important in Uganda, and why?	• What do you believe are the main reasons to support the introduction of guidelines or other general recommendations for managing wastewater, septage, or sludge?<br>• What do you believe are the main reasons to oppose the introduction of guidelines for managing wastewater, septage, or sludge?<br>• Which technical or feasibility arguments do you believe to be of relevance when opposing or supporting the introduction of guidelines for managing wastewater, septage, or sludge?<br>• What economic or financial considerations do you believe to be of relevance when opposing or supporting the introduction of guidelines for managing wastewater, septage, or sludge?<br>• Which social and cultural aspects do you take into account when you support or oppose the introduction of guidelines for managing wastewater, septage, or sludge?<br>• What ethical arguments are reflected when considering the support or the opposition of the introduction of guidelines for managing wastewater, septage, or sludge?<br>• What role do the results from scientific research play in the considerations on the support or the opposition of the introduction of guidelines for managing wastewater, septage, or sludge?	Assess what participants perceive as relevant criteria for decision making
**3**	What role do WHO guidelines play in national decision-making processes on water, sanitation and hygiene?	• What role do the recommendations in WHO Guidelines play in national decision-making processes in the area of water, sanitation and hygiene?	Assess how participants evaluate the usefulness of WHO guideline recommendations

**Adapted EtD framework**<br>• Good time for a restroom/refreshment break. After allowing a a short time to review EtD document individually, suggest facilitator gives another short introduction to reorient group discussion around the EtD (ours got a little stuck on discussing the sanitation guidelines)			
**6**	Provide the participants with the adapted EtD framework and invite them to read it thoroughly.<br>Please, tell me about your first impression of this framework.	• In what way does the framework reflect your thoughts and the general discussion about managing wastewater, septage, or sludge?	Assess the first impression of the participants of the revised EtD framework
	What is your opinion about the overall structure of the framework?	• How do you rate the relevance of the health benefits aspects?<br>• To what extent are the acceptability aspects of relevance?<br>• To what extent are the equity aspects of relevance?<br>• How do you rate the relevance of the long-term social, economic and environmental benefits aspects?<br>• To what extent are the utility aspects of relevance?<br>• To what extent are the feasibility aspects of relevance?	Assess how the participants evaluate the overall structure of the adapted EtD frameworks
**7**	What is your opinion about the criteria and sub-criteria in the framework?	• Are any criteria or sub-criteria missing from the adapted EtD framework? If so, which ones?<br>• Are any criteria or sub-criteria not needed in the framework? If so, which ones?	Assess how the participants evaluate the criteria in the adapted EtD frameworks
**8**	Do you think this framework is useful when thinking about the introduction of guidelines for managing wastewater, septage, or sludge?	• How do you think is the understandability of this framework?<br>• How do you feel about the ease of use of the framework when thinking about introducing public health interventions?<br>• What is your opinion on the applicability of the framework at national, district or program level (your specific needs)?	Assess how participants evaluate the adapted EtD frameworks in terms of practical considerations
**8**	Do you believe a WHO guideline taking the criteria of the framework into account would be useful for your decision-making process? Why?	• Do you think these criteria are a useful basis for guidelines adapted or developed in Uganda on managing wastewater, septage, or sludge? Why?<br>• Do you think these criteria are a useful basis for other public health guidelines adapted or developed in Uganda? Why?	Assess whether the participants perceive the framework as useful/not useful
Interviewer Instructions			
• Thank again for the time and willingness to participate in the FGD.<br>• Ask if there are any questions left and answer all questions.<br>• Stop the tape and say goodbye.			
