# Supplementary material for: Advancing the WHO-INTEGRATE Framework as a Tool for Evidence-Informed, Deliberative Decision-Making Processes: Exploring the Views of Developers and Users of WHO Guidelines
Source: Int J Health Policy Manag. 2020 Oct 27;11(5):629–41. doi: 10.34172/ijhpm.2020.193 (PMC9309924; doi:10.34172/ijhpm.2020.193)
Supplement: Supplementary file 2 — Details on KIIs and FGDs. [file ijhpm-11-629-s002.pdf]

Supplementary file 2. Details on Key Informant Interviews and Focus Group Discussions

1.1 Additional information on key informant interviews

The interviewees were approached directly by the researchers (JMS, EAR) through the WHO Guidelines Review Committee (GRC) or through other WHO intermediaries. Interviews were conducted in English by a male researcher (JMS) with experience in qualitative research and familiar with the WHO-INTEGRATE framework and the topic of the guideline[1] [2]. The participants were informed about the aim of the study and the interviewer's background and involvement in the development of the WHO-INTEGRATE framework.

We conducted n=9 KIIs (WHO staff coordinating the guideline: n=2, GDG chair: n=4, methodologist: n=3; female: n=7). In three cases, the interviews were conducted face-to-face at the WHO headquarters in Geneva; in six cases the interviews were conducted via telephone or video call. The interviews had a median duration of 62 minutes (57–69 minutes) and were conducted between June and October 2017. Two interviews did not take place as the participants had retired, and one interview could not be arranged despite several contact attempts.

1.2 Additional information on focus group discussions

Supplement table S1.1: Countries, thematic areas and topics of focus group discussions

**Country**	**Brazil**	**Germany**	**Nepal**	**Uganda**
Researcher(s) conducting FGD	AAM, CEMR	JMS	DP	JO; KS
Presence of non-participants	JMS (remotely from Germany)<br>Two student assistants were present, acting as administrative and logistical support for AAM and CEMR.	None	JMS	One observer from national WHO office
Researcher's credentials	AAM: PhD<br>CEMR: MD, PhD	JMS: MD, B.Sc.	DP: PhD, MPH<br>JMS: MD, B.Sc.	JO: BEHS, MSc<br>KS: MESM, B.Sc.
Researcher's occupation	AAM: Professor, Department Maternal-Infant Nursing and Public Health University of São Paulo<br>CEMR: Health insurance system regulatory agency, Brazil	JMS: Doctoral student, Pettenkofer School of Public Health, LMU Munich	DP: Health Director, Save the Children<br>JMS: Doctoral student, Pettenkofer School of Public Health, LMU Munich	JO: Research Associate, School of Public Health, Makerere University,<br>KS: Doctoral student, Gillings School of Global Public Health, University of North Carolina at Chapel Hill
Gender	Male: CEMR<br>Female: AAM	Male: JMS	Male: DP & JMS	Male: JO, JSS<br>Female: KS
What information did participants have prior to participation?	The participants were informed about the aim of the study, its contribution to the overall research project and the moderators' professional background and involvement (if applicable) in development of the WHO-INTEGRATE framework.			
How were participants recruited?	In Brazil, we primarily recruited from the members of the national tuberculosis control program responsible for reviewing, analysing, adapting and implementing WHO guidelines, which comprises representatives from all five major macro-regions of Brazil.	In Germany, we recruited participants through the Bavarian Health and Food Safety Authority.	In Nepal, the main entry-point for identification and recruitment was a technical working group on maternal and child health, chaired by the Ministry of Health and Population, which includes representatives from the Ministry, donors, technical assistance organizations, academia and non-governmental organizations.	In Uganda, two main entry-points for participant identification and recruitment were pursued, i.e. the national water and sanitation committee and the Ugandan country representatives in the global Sanitation and Water for All partnership.
Provision of supplemental information	CEMR gave a short presentation on the framework between the two phases of the FGD.	One participant gave a short introduction of the evidence of the health effects of high-fructose corn syrup, which concluded that there is uncertainty as to whether high-fructose corn syrup poses a health risk.	JMS gave a short presentation on the framework between the two phases of the FGD.	No presentation was given during the FGD.
