# Supplementary material for: Advancing the WHO-INTEGRATE Framework as a Tool for Evidence-Informed, Deliberative Decision-Making Processes: Exploring the Views of Developers and Users of WHO Guidelines
Source: Int J Health Policy Manag. 2020 Oct 27;11(5):629–41. doi: 10.34172/ijhpm.2020.193 (PMC9309924; doi:10.34172/ijhpm.2020.193)

**Supplementary file 3.** Coding Frames Used to Code Transcripts of Key Informant Interviews and Focus Group Discussions

**Supplement Figure S3.1**: Coding frame for coding the KII transcripts

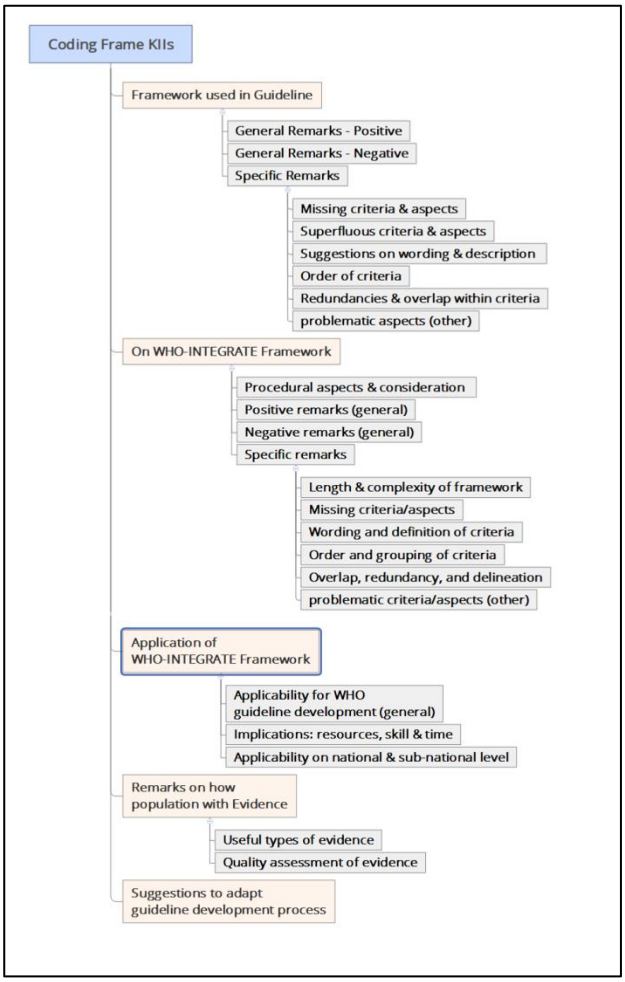


**Supplement Figure S3.2**: Coding frame for coding the FGD transcripts

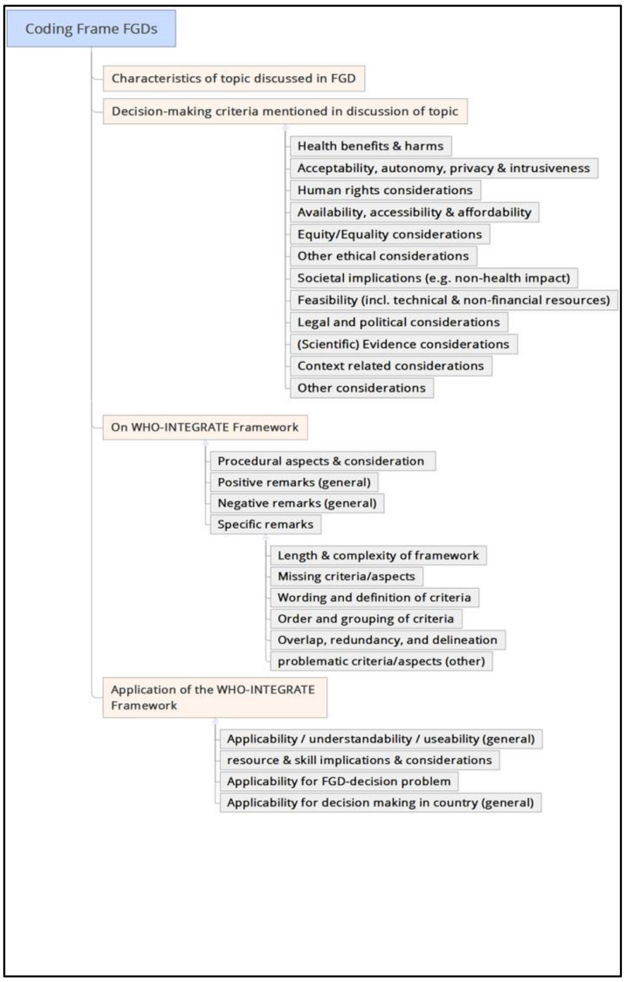

Supplement: Supplementary file 3 — Coding Frames Used to Code Transcripts of KIIs and FGDs. [file ijhpm-11-629-s003.pdf]
