# Supplementary material for: Advancing the WHO-INTEGRATE Framework as a Tool for Evidence-Informed, Deliberative Decision-Making Processes: Exploring the Views of Developers and Users of WHO Guidelines
Source: Int J Health Policy Manag. 2020 Oct 27;11(5):629–41. doi: 10.34172/ijhpm.2020.193 (PMC9309924; doi:10.34172/ijhpm.2020.193)
Supplement: Supplementary file 4 — Suggested Changes to the WHO-INTEGRATE Framework Based on KIIs and FGDs. [file ijhpm-11-629-s004.pdf]

Supplementary file 4. Suggested Changes to the WHO-INTEGRATE Framework Based on Key Informant Interviews and Focus Group Discussions

Supplement Table S4.1: Text examples of decision-making aspects and considerations discussed in the respective Focus Group Discussions (FGDs) in the Country Case Studies (CCS) in Brazil, Germany, Nepal and Uganda set in relation to those criteria and sub-criteria in the WHO INTEGRATE Framework.

**Criteria (shaded rows) & Sub-Criteria**	**KII- suggestions by experts**	**Considerations by framework developers based on CCS FGDs & KIIs**	**CCS FGDs: Suggestions by participants**
**Balance of health benefits and harms**		• **Missing criterion or aspect / Wording and definition**: Consider whether the broad concept of health (according to WHO definition) is adequately emphasized in this criterion	
**Efficacy or effectiveness on health of individuals**		• **Missing criterion or aspect / Wording and definition**: Consider whether aspects of individual preferences (wellbeing and personal values and preferences regarding health and life) is adequately emphasized in this criterion	• **Missing criterion or aspect / Wording and definition**: Consider whether aspects of individual preferences (wellbeing and personal values and preferences regarding health and life) is adequately emphasized in this criterion
**Patients'/beneficiaries' values in relation to health outcomes**	• **Order and grouping of criteria:** Consider values and preferences as a main criterion, rather than a sub-criterion to give it adequate weight<br>• **Wording and definition // Overlap, redundancy and delineation:** Consider whether the criterion is adequately described and whether an expansion on the criterion is needed. Consider a potential overlap with sub-criterion related to acceptability.	**Wording and definition**: Consider, rewording criterion "values and preferences" to avoid confusion with differently focused criterion in the DECIDE EtD framework	• **Wording and definition:** increase understandability of criterion (general)
**Safety-risk-profile of intervention**			• **Wording and definition:** Consider whether the breadth of the concept of risk & harm is adequately expressed in definition.
**Broader positive or negative health-related impacts**		• **Wording and definition // Overlap, redundancy and delineation:** Consider whether the breadth of the concept is adequately expressed in definition and whether the delineation to the other criteria relating to health benefits and harm is adequate.	

**Human rights and socio-cultural acceptability**		**• Order and grouping of criteria:** Consider separating "human rights" and "socio-cultural acceptability in two different criteria.	**• Order and grouping of criteria:** Consider separating "human rights" and "socio-cultural acceptability in two different criteria.<br>**• Missing criterion or aspect:** Consider, whether the consideration of vulnerable, marginalized and/or underserved populations is adequately reflected in the criterion and if there is a need for strengthening this aspect.
**Accordance with universal human rights standards**			**Wording and definition:** Consider whether the description and definition of criterion is adequate and whether the focus on issues beyond the right to health is adequately covered.
**Socio-cultural acceptability of intervention to patients/ beneficiaries and those implementing the intervention**	**• Overlap, redundancy and delineation // Missing criterion or aspect: A**cceptability can be regarded as value in itself, as a perquisite for feasibility and/or as a building block for impact (eg, adherence). Consider whether or not these aspects are covered and / or adequately delineated.<br>**• Wording and definition // Overlap, redundancy and delineation:** Consider whether the criterion is adequately described and whether an expansion on the criterion is needed. Consider a potential overlap with sub-criterion "Patients'/beneficiaries' values in relation to health outcomes"	**• Overlap, redundancy and delineation:** Consider possible overlap and adequate delineation of sub-criterion and "impact on autonomy of concerned stakeholders"<br>**• Overlap, redundancy and delineation // Missing criterion or aspect: A**cceptability can be regarded as value in itself, as a perquisite for feasibility and/or as a building block for impact (eg, adherence). Consider whether or not these aspects are covered and / or adequately delineated.	**• Wording of definition // assess the need for practical guidance in framework usage:** how to handle acceptability as a dynamic aspect prone to change and external influence.
**Socio-cultural acceptability of intervention to the public and other relevant stakeholder groups**	**• Wording of definition // assess the need for practical guidance in framework usage:** Consider providing more clarification and guidance, especially in regards to which stakeholders are meant to be included and how to handle the plurality of positions held by various ethno-cultural communities and other stakeholders in regards to the various guideline topics		**• Reassess wording & definition // Missing criterion or aspect:** Consider whether family as a stakeholder group is adequately covered and whether it should be made more explicit in the definition.
**Impact on autonomy of concerned stakeholders**			
**Intrusiveness of intervention**		**• Wording and definition:** Consider whether the criterion is adequately and clear enough described to avoid misunderstandings and erroneous interpretations.	**• Missing criterion or aspect**: Consider, whether the criterion needs to be expanded to adequately cover the issue of liberty/freedom (German: Freiheit)<br>**• Wording and definition:** Consider whether the criterion is adequately and clear enough described.

**Equity, Equality and Non-Discrimination**	• **Missing criterion or aspect:** Consider, whether the consideration of vulnerable, marginalized and/or underserved populations is adequately reflected in the criterion “health equity, equality and non-discrimination” and a need for strengthening this aspect.	• **Wording and definition // missing criteria and aspects:** consider whether issues of gender, age, and geography are adequately covered in criterion regarding particular needs and distribution of outcomes.	• **Order and grouping of criteria:** Consider whether non-discrimination should be reflected alongside with the human rights considerations, rather than under the criterion Equity, equality and non-discrimination.<br>• **Wording and definition // Missing criterion or aspect:** consider whether issues of gender equity and/or discrimination based on gender are adequately covered in criterion<br><br>• **Wording and definition // Missing criterion or aspect:** consider whether the different needs of different populations are adequately reflected in framework
**Distribution of benefits and harms of intervention**		• **Wording and definition // Missing criterion or aspect:** consider whether issues of gender, age, and geography are adequately covered in criterion regarding particular needs and distribution of outcomes.	
**Affordability of intervention**	• **Wording and definition // Overlap, redundancy and delineation:** Assess criterion title and definition and consider, whether a rewording would be helpful in order to emphasize the focus on individuals and on the issue of catastrophic health expenditures.		• **Order and grouping of criteria:** consider placing this sub-criterion either to criterion societal implications or to financial and economic considerations
**Accessibility of intervention**		• **Wording and definition:** Assess whether the wording and is adequate for (complex) public health interventions (eg, is informational accessibility to labeling adequately covered and comprehensible?)	
**Lack of a suitable alternative**		• **Wording and definition // Overlap, redundancy and delineation:** Assess whether the wording and is adequate for (complex) public health interventions (eg, should limited effectiveness of an intervention based on providing information be considered lack of a suitable alternative?)	

**Societal Implications**	• **Wording and definition:** Consider whether the criterion is adequately described and whether an expansion on the criterion is needed.<br>• **Assess the need for practical guidance in framework usage:** Within guideline development, as the evidence on this criterion could be the same for several recommendations covering a similar topic within one guideline, one could consider providing an overarching consideration of "societal implications" for multiple recommendations in the final guideline document.	• **Wording and definition:** Consider whether the criterion is adequately described and whether an expansion on the criterion is needed.<br>• **Wording and definition // Overlap, redundancy and delineation:** Consider whether the delineation between health impact is clear and adequate.	• **Wording and definition:** Consider whether the criterion is adequately described and whether an expansion on the criterion is needed.<br>• **Order and grouping of criteria:** Consider whether societal impact and "health impact" should be considered within one criterion or alongside with each other.
**Social impact**	• **Overlap, redundancy and delineation:** Assess a potential overlap between criterion "health equity, equality and non-discrimination" and the sub-criterion of "social impact", as reduced inequity could be regarded as a social impact. Consider the need for improving discription, definition and/or delineation.<br>• **Wording and definition:** Consider whether the term implications rather than impact would be more appropriate for the criterion.	• **Wording and definition:** Consider whether the criterion is adequately described and whether an expansion on the criterion is needed.	
**Environmental impact**	• **Wording and definition:** Consider whether the term implications rather than impact would be more appropriate for the criterion.	• **Wording and definition:** Consider whether the criterion is adequately described and whether an expansion on the criterion is needed.	• **Wording and definition:** Consider whether the criterion is adequately described and whether an expansion on the criterion is needed.

**Financial and Economic Considerations**		• **Wording and definition:** consider whether the definition adequately reflects a considerations of different payers within and beyond the health care system and individual (reflect on issue of cost shifting) on all levels of the implementation chain of an intervention.	
**Financial impact**	• **Wording and definition:** Consider whether the term implications rather than impact would be more appropriate for the criterion.	• **Reassess wording & definition // Missing criterion or aspect:** Consider whether the criterion adequately reflects the heterogeneity of monetary resources available on all links of the implementation chain of an intervention.	• **Redundancy and overlap:** Financial considerations, economic considerations and affordability of intervention can intermingle and overlapping (eg, in cases of different payers at different stages of disorder). Consider, whether delineation and distinction are adequate.
**Impact on economy**		**Wording and definition:** Consider whether the criterion is adequately described and whether an expansion on the criterion is needed.	
**Ratio of costs and benefits**			• **Wording and definition // consider criterion reported as missing:** criterion of cost-effectiveness ratios was reported as missing by participants

Feasibility and Health System considerations			
**Legislation**		• **Redundancy and overlap:** Consider whether legal and political feasibility is adequately delineated.<br>• **Wording and definition:** Consider whether the criterion is adequately described and whether an expansion on the criterion is needed.	• **Redundancy and overlap:** Consider whether legal and political feasibility is adequately delineated.
**Leadership and governance**		• **Missing criterion or aspect:** consider, whether political considerations (eg, in the form of lobbyism should be considered more explicitly (eg, as part of a criterion of political feasibility); at least, to make it an explicit, rather than an implicit for of influence.	• **Missing criterion or aspect // wording and definition**: Consider, whether political and administrative feasibility (barriers and facilitators) are adequately captured and described in criterion<br>• **Missing criterion or aspect:** consider, whether political considerations (eg, in the form of lobbyism should be considered more explicitly (eg, as part of a criterion of political feasibility).
**Interaction with and impact on health system**	**Overlap, redundancy and delineation**: Assess a potential overlap between the sub-criterion “Interaction with and impact on health system " and the criterion “financial and economic considerations”.	• **Reassess wording & definition // Missing criterion or aspect:** Consider whether the criterion is adequately described to emphasize the broad understanding of health system and/or whether an aspect of intersectoral cooperation is missing.	• **Wording and definition // consider criterion reported as missing:** dependency on activity & action of other elements of the health system as a basis for intervention implementation and functioning<br>• **Reassess wording & definition // Missing criterion or aspect:** Consider whether the criterion is adequately described and whether an expansion on the criterion is needed, as participants noted that integration of intervention in existing (health) system was missing.<br>• **Reassess wording & definition // Missing criterion or aspect:** Consider whether the criterion is adequately described to emphasize the broad understanding of health system and/or whether an aspect of intersectoral cooperation is missing.
**Need for, usage of and impact on health workforce and human resources**			• **Reassess wording & definition // Missing criterion or aspect:** Consider whether the criterion adequately reflects the heterogeneity of infrastructure availability on all links of the implementation chain of an intervention

**Need for, usage of and impact on infrastructure**		• **Wording and definition:** Consider whether the criterion is adequately described and whether an expansion on the criterion is needed to improve broad understanding of "infrastructure" in the sense of the criterion.<br>• **Reassess wording & definition // Missing criterion or aspect:** Consider whether the criterion adequately reflects not only the issue of currently available infrastructure, but also the issue of availability, implementability and procurement of needed infrastructure<br>• **Reassess wording & definition // Missing criterion or aspect:** Consider whether the criterion adequately reflects the heterogeneity of infrastructure availability on all links of the implementation chain of an intervention.	• **Wording and definition // consider criterion reported as missing**: availability and capacity of institutions and structures for planning, monitoring, and evaluation of intervention

**Missing criteria**		• **Missing criterion or aspect:** consider, whether political considerations (eg, in the form of lobbyism should be considered more explicitly (eg, as part of a criterion of political feasibility); if only to make these influences explicit rather than implicit.<br><br>• **Missing criterion or aspect:** Consider whether the sustainability and life-course perspective regarding feasibility (maintenance, repairs etc.) is adequately reflected or if a new criterion should be added.<br><br>• **Missing criterion or aspect:** Consider whether the dimension of time is adequately covered ; eg, time until impact is achieved (where sooner would be better than later)	• **Missing criterion or aspect:** Assess whether the sub-criterion reported as missing truly is missing and/or should be added to the framework: Sustainability of intervention / recommendation<br><br>•**Missing criterion or aspect:** consider, whether political considerations (eg, in the form of lobbyism should be considered more explicitly (eg, as part of a criterion of political feasibility).<br><br>• **Missing criterion or aspect:** consider, whether a criterion regarding multi-sectoral collaboration is adequately captured.<br><br>• **Missing criterion or aspect:** Consider whether the issue of reliability and quality of an intervention is adequately covered in the framework or needs to be added.
**Order of criteria in general**			• **Order and grouping of criteria:** Consider whether societal impact and "health impact" should be considered within one criterion or alongside with each other.<br><br>• **Order and grouping of criteria:** Balance of health benefits and harms should be the first criterion, followed by the "feasibility-oriented" criteria, sub-criteria, and aspects financial and economic considerations and health systems and feasibility considerations; all other criteria, sub-criteria and aspects should be considered after that.<br><br>• **Order and grouping of criteria:** Consider whether "health systems considerations" should be moved to a higher position within the framework to emphasize the criterion's importance.
