# Supplementary material for: Advancing the WHO-INTEGRATE Framework as a Tool for Evidence-Informed, Deliberative Decision-Making Processes: Exploring the Views of Developers and Users of WHO Guidelines
Source: Int J Health Policy Manag. 2020 Oct 27;11(5):629–41. doi: 10.34172/ijhpm.2020.193 (PMC9309924; doi:10.34172/ijhpm.2020.193)
Supplement: Supplementary file 5 — Suggested Changes to the WHO-INTEGRATE Framework Based on FGDs With Exemplary Quotes. [file ijhpm-11-629-s005.pdf]

Supplementary file 5. Suggested Changes to the Who-INTEGRATE Framework Based on FGDs With Exemplary Quotes

Supplement table S5.1: Text examples of decision-making criteria discussed in the respective Focus Group Discussions (FGDs) in the Country Case Studies (CCS) in Brazil, Germany, Nepal and Uganda set in relation to those criteria in the WHO INTEGRATE Framework.

Criterion	Sub-Criterion	CCS FGD Brazil	CCS FGD Germany	CCS FGD Nepal	CCS FGD Uganda
	**Balance of health benefits and harms**			• **Missing criterion or aspect / Wording and definition**: Consider whether aspects of individual preferences (wellbeing and personal values and preferences regarding health and life) is adequately emphasized in this criterion:<br>*The benefits as we define as public health and medical professional and the benefits that adolescent would define in that age is different. The pleasure of being together with a partner, physical contacts, enjoying beer and cigarette for them is special. I am not sure if that benefits is considered and if here or somewhere else*	
	**Efficacy or effectiveness on health of individuals**				
	**Effectiveness or impact on health of population**				
	**Patients'/beneficiaries' values in relation to health outcomes**	**Re-assess wording and definition**<br>"Just an explanation, this subcriterion: values of patients, population, the value here means…"			

	**Safety-risk-profile of intervention**	**Reassess wording & definition: increase understandability of criterion (general)**<br>F: No, it's this pondering the risk it's just treatment risk, is that right?<br>H: Not necessarily, because we're discussing it here in a global way, the development of an instrument that can be used in the future, not just in the incorporation of a technology, but public policy analysis too.			
	**Broader positive or negative health-related impacts**				
	**Human rights and socio-cultural acceptability**	**• Consider separating "human rights" and "socio-cultural acceptability in two different criteria.**<br>*P1: I can't see the two together, for me they're two things, one is talking about human rights specifically the question of freedom, etc., and the other is talking about socio-cultural acceptability because not necessarily the acceptability I'm violating, cultural acceptability I'm meeting human rights, because then it's a matter of country, a matter of culture, it's in another context. […] The separation would be something really important here in this situation, I see two things. Even by the description that is here it assumes there are two things, as it describes, it can't put human rights and acceptability together as if they were only one thing, it addresses the topic as if there were two things, so if it's two, it's two criteria and not a mix.*			

	**Accordance with universal human rights standards**	**Wording & Definition: Consider whether the description and definition of criterion is adequate and whether the focus on issues beyond the right to health is adequately covered.**<br>*H: Yes, many human rights that I cited there was just one of the subcriteria on human rights, I think a reinforcement to health is pertinent indeed, something making it clearer. It's a lot, I even understand that according to the human rights universal standards health is included in these human rights, but in a WHO document not to emphasize right to health, and then not necessarily associated with human rights, but to something specific of health, etc., to the citizen, to the person, it's something that would be missing, so I had thought of this and with the discussions I forgot, so [NAME] reminded me that it was necessary to let this well characterized.*<br>*F: Indeed, this is really important, when there is this criterion the population deprived of freedom mostly, that we already have a stigma, a difficulty to understand the population has right to health specifically, and then the municipality itself to understand it needs to incorporate that care in the municipal network and everything else, so yes.*			
	**Socio-cultural acceptability of intervention to patients/ beneficiaries and those**	**• Overlap, redundancy and delineation // Missing criterion or aspect:**<br>Acceptability can be regarded as value in itself, as a perquisite for feasibility and/or as a building block for impact (eg, adherence). Consider whether or not			

	**implementing the intervention**	these aspects are covered and / or adequately delineated.<br>*For instance, now we're trying to bring the fixed pediatric dose, which we don't use in Brazil and would help, we believe it'd increase adherence, because at present we work with recommendations like that, we have two presentations of the main drugs for children, but not for the other medicines, then we have to, the recommendation is to macerate the pill so the child can swallow it. So I think this is on stand three of the plan and will help a lot in this struggle which is to try to improve the treatment and eradicate TB as a public health problem until 2035. So we're working internally for this to happen, together with other areas of the ministry.*<br><br>**Consider rewording of definition // assess the need for practical guidance in framework usage: how to handle acceptability as a dynamic aspect prone to change and external influence.**<br>"Yes, I got it well [NAME], I think acceptability is fine, it has to come from the person, to accept a treatment, but the persuasion is not by the person, the persuasion is, comes first from […] the healthcare professional. So, it's him or her who has, the person wants to get well, the person doesn't want, he or she is going to take the medication if convinced that the medication is going to heal him or her, it doesn't matter. I find it hard to take an antibiotic for 14 days, whatever it is. I wonder how it is when you have to take six, so it has to be persuasion, so it			

		originates from the person's acceptance, right? But the persuasion, the approach makes the difference too."			
	**Socio-cultural acceptability of intervention to the public and other relevant stakeholder groups**			• **Reassess wording & definition // consider missing sub-criterion:** Consider whether family as a stakeholder group is adequately covered and whether it should be made more explicit in the definition.<br>*Interviewer: Are any criteria or sub-criteria missing from the adapted EtD framework? If so, which ones? Are any criteria or sub-criteria not needed in the framework? If so, which ones?*<br>*P1: We may consider family within the "relevant stakeholder group" but it should be more explicit*	
	**Impact on autonomy of concerned stakeholders**	**Consider (sub-)criterion in the light of a challenge: autonomy and reduced acceptability in the light of limited and erroneous knowledge regarding the subject.**<br>*[...] These two items there, the impact on the autonomy of the involved interested parts and intrusiveness in the intervention, there is an aspect [...] which is very technical [...]. [...] Yes, to what extent does this possibility of acceptance go considering that we can't measure the knowledge they have on the subject to oppose it, or even of the patient, because there were sporadic, rare reports of patients who were bothered, who had already done the exam and didn't know what it was, and wanted another one. So, it's everyone's right in the health system to have the care that he or she considers the best one. But to what extent does this limit of divergence go when you have*			

		well-defined, well-established technical criteria and from these criteria you establish a change in what was established before? Got it? [...] the person comes in the service and says "I don't want this, I want another service, I want the other test", or the person says "no, I read an article saying this new four-in-one medicine is killing more people, I don't want the four-in-one, I want them all separated". When all the evidence and scientific studies and organizations point to the contrary, so I think this is a very sensitive subject to all the involved parts, so I don't know if there is a limit up to which you consider this or you don't.			
	**Intrusiveness of intervention**		**Missing criterion or aspect**: Consider, whether the criterion needs to be expanded to adequately cover the issue of liberty/freedom (German: Freiheit)<br>*"And the criterion that nominally opposes the whole thing here and what I have missed in this explicit form, so to speak, is the concept of freedom. Because that which is circumcised is ultimately individual freedom, the freedom of society. And now again, when you think about statehood, that is of course actually a core concept around which everything revolves."*	• **Wording and definition:** Consider whether the criterion is adequately and clear enough described.<br>*Can you please explain what intrusiveness means?*	• **Wording and definition:** Consider whether the criterion is adequately and clear enough described to avoid misunderstandings.<br>*I think it- the language comes a little bit more from the medical care field, um, where a test or, um, or a vaccination might be painful. There might be some negative consequences. So, you wanna make sure it's not something very intrusive that people would say, "oh no, me personally, I don't want to use that because, you know, it would be harmful to me." So that's the explanation, if it makes sense.*
	**Equity, Equality and Non-Discrimination**	**Consider, whether non-discrimination should be reflected alongside with the human rights considerations, rather than under the criterion Equity, equality and non-discrimination.**<br>*F: Just to stress what [NAME] commented, I think in the text this part in which*		• **Wording and definition // missing criteria and aspects:** consider whether the different needs of different populations are adequately reflected in framework.<br>*In some cases, we have not been gender sensitive and gender balanced. We are assigning young fresh graduate ANMs to*	**Wording and definition // missing criteria and aspects:** consider whether issues of gender equity are adequately covered in criterion<br>*I was just wondering. So, in some books you will find it is written, gender equality,*

		equality is associated with the legal principle of non-discrimination, I'd say something like equality is associated with the legal principle of right to health, not discriminating doesn't mean the person is going to have access, like oh I'm not discriminating, but I don't want everybody to have access to health. It doesn't mean everybody has to be rich.<br><br>"Personally I don't like the word non-discrimination, if equality implies this non-discrimination, when I say non-discrimination I emphasize the discrimination, I call it into the discussion, so health equity and equality. This is my criticism regarding the criterion, and it's obvious I'd also change the description of what it says here that equality is related to a legal principle of non-discrimination, so equality is assured that people () etc., etc., would follow, I wouldn't address either discrimination or non-discrimination in this case for finding it unnecessary taking into account the concept of equality. And from the point of view of using this criterion for decision-making by prioritizing certain policies, technological incorporations."		*deliver ASRH services in the health facility.Can young boys seek services from them? We found that adolescent boys are hesitants to seek services from them. We have not been able to focus on such matters. Guideline should consider these aspects.*<br><br>**Wording and definition // missing criteria and aspects:** consider whether issues of gender equity are adequately covered in criterion<br><br>*"P4: Where does gender equality included?*<br>*P5: I prefer to keep that somewhere*<br>*P6: That is the part of societal impact*<br>*P4: Here it talks about ethical and socio-cultural acceptability and gender doesn't fit well here*<br>*P5: In gender, we have left boys at all. Except the advertisement in the Kantipur (national TV channel), there is nothing focusing them*<br>*Interviewer: So, your suggestions are to keep gender more explicitly and it should not be understood as focusing girls but balancing between boys and girls as per the need.*<br>*Multiple: Yes, that is what we meant*<br>*P4: We talk about representational approach but all ASRH services are girl focused, and there is nothing about boys. We need to consider that"*<br><br>**Wording and definition // missing criteria and aspects:** consider whether issues of gender, age, and geography are adequately covered in criterion regarding	*nondiscrimination, it is an issue. So, in this one, gender equality.*

				particular needs and distribution of outcomes.<br><br>*Should we consider gender, age and geography somewhere?*	
	**Impact on health equality and/or health equity**				
	**Distribution of benefits and harms of intervention**			**Wording and definition // missing criteria and aspects:** consider whether the different needs of different populations are adequately reflected in framework<br><br>*"P4: Where does gender equality included?*<br>*P5: I prefer to keep that somewhere*<br>*P6: That is the part of societal impact*<br>*P4: Here it talks about ethical and socio-cultural acceptability and gender doesn't fit well here*<br>*P5: In gender, we have left boys at all. Except the advertisement in the Kantipur (national*<br>*TV channel), there is nothing focusing them*<br>*Interviewer: So, your suggestions are to keep gender more explicitly and it should not be understood as focusing girls but balancing between boys and girls as per the need.*<br>*Multiple: Yes, that is what we meant*<br>*P4: We talk about representational approach but all ASRH services are girl focused, and there is nothing about boys. We need to consider that"*	

	**Affordability of intervention**	**Order & position: consider placing this sub-criterion either to criterion societal implications or to financial and economic considerations**<br>*H: So, I'm not sure if I heard well what [Name] said, but maybe it's the case of bringing the financial part which is in the item, in the previous criterion, it's addressing population, addressing people, to address financial and economic conditions and put what is related to management or budget as it says here, and even the cost of the person, maybe mentioning two places of resources, of costs, etc. is a complicating factor when we are going to evaluate, because sometimes they mix up, it we mention money, what we spend and what it represents to the population too, to the person who has, I don't know, perhaps it's…*			
	**Accessibility of intervention**				
	**Severity and/or rarity of the condition**				
	**Lack of a suitable alternative**				
	**Societal Implications**	**Wording & Definition:** Consider whether the criterion is adequately described and whether an expansion on the criterion is needed.<br>*From my point of view, to me it isn't clear what this criterion wants. I couldn't understand, think in an operational way, but taking a guess from the little I understood, perhaps the idea of the new ILTB for patients with () 350, is a new*		**Wording & Definition:** Consider whether the criterion is adequately described and whether an expansion on the criterion is needed.<br>*"P4: Where does gender equality included?*<br>*P5: I prefer to keep that somewhere*<br>*P6: That is the part of societal impact*<br>*P4: Here it talks about ethical and socio-cultural acceptability and gender doesn't*	

		recommendation that has social, environmental, economic impacts, and it's still a new standard. So maybe, thinking of all that the social implications, for health, for the network, that this would have, maybe this could be an example, I don't know if I made myself clear.		*fit well here*<br>*P5: In gender, we have left boys at all. Except the advertisement in the Kantipur (national*<br>*TV channel), there is nothing focusing them*<br>*Interviewer: So, your suggestions are to keep gender more explicitly and it should not be understood as focusing girls but balancing between boys and girls as per the need.*<br>*Multiple: Yes, that is what we meant*<br>*P4: We talk about representational approach but all ASRH services are girl focused, and there is nothing about boys. We need to consider that"*<br><br>• **Wording and definition // Overlap, redundancy and delineation:** Consider whether the delineation between health impact is clear and adequate.<br><br>*P4: Where do we consider 'impact' in this new framework? Other May be as part of "societal impact"*<br>*P5: No, health impact and social impact are different things.*<br>*Interviewer: In my view, it is included as part of "balance of health benefits" Impact is about health benefits. Other broader benefits are in societal impacts. If you have any suggestions on what might be the right terminology, you can share that too*	
	**Social impact**	**Wording & Definition:** Consider whether the criterion is adequately described and whether an expansion on the criterion is needed.<br><br>*So let's get to the explanation. The impact here, it doesn't necessarily has to do with values, it's impact in terms of*			

		improvement, non-improvement, worsening of the population's health from an intervention, it's in this sense that this social impact is here.			
	**Environmental impact**			**Wording & Definition:** Consider whether the criterion is adequately described and whether an expansion on the criterion is needed.<br><br>*The terminology on "environmental impact" is a bit vague. In societal impact there are enough questions to help understand but in environmental there is just a question on climate change. So, it needs to be expanded.*	**Wording & Definition:** Consider whether the criterion is adequately described and whether an expansion on the criterion is needed.<br><br>*Much as comes in environment should come out specifically because it's a big issue. And it has caused us it has added climate change. It is a disaster, [inaudible]? So, it should come out on environmental impact... in your text, should come out clearly.*
**Financial and Economic Considerations**	**General**				
	**Financial impact**	**Redundancy / Overlap: Financial considerations, economic considerations and affordability of intervention can intermingle and overlapping (eg, in cases of different payers at different stages of disorder). Consider, whether delineation and distinction is adequate.**<br><br>*P1: Also because of the catastrophic costs that are already calculated, got it? When the person…*<br>*P2: So, I have a doubt, [NAME] can, because [NAME] already anticipated it and brought up the resource issue, so he can discuss this better than me, I'm following the order here. So I didn't even want to mention the catastrophic cost thing yet, but we can talk about, how much it costs when the person gets sick, what is lost, what is won. Not winning,*			

		nothing is won, of course…<br>*P1: I think that in the future, where [NAME] pointed to, it's more about the cost to the management, and this cost has more to do with the cost to the person, the social one, right?*<br>*P3: I see this question, what happens at the end of it, until that decision issue really, what are we going to do… For instance, it's much cheaper to treat a new case than a resistant one, so this is going to be better focused on (), so in the decision-making process, what I said that I think the financial point that is there in the…*			
	**Impact on economy**				
	**Ratio of costs and benefits**	**Consider increasing visibility of criterion**<br>"[..] it seems to be that the subcriteria lack a cost-benefit ratio, I don't know if it should be included here, it's a financial impact, it's an impact on the economy, costs and benefits and it seems to me that there's still cost-benefit there to see…"			
**Feasibility and Health System considerations**					
	**Legislation**				
	**Leadership and governance**			**Missing criterion or aspect // wording and definition**: Consider, whether political and administrative feasibility is adequately captured and described in criterion<br>*"P1: While reviewing all criteria and sub-criteria, it is very comprehensive. I think, we should specific one thing here. In the "feasibility and health system consideration", there is a sub-criteria on 'legislation and governance" and it*	**Missing criterion or aspect // wording and definition**: Consider, whether political and administrative feasibility (barriers and facilitators) are adequately captured and described in criterion<br>*P1: I also wanted, didn't see clearly the political, the political aspects. What- how it impacts on all this. Whether it's implied in-in the legal governance barriers or facilitators, not quite clear. So, from experience here, the political situation*

				captures legal barriers and facilitators. I think, "ability to execute a policy" which might include administrative feasibility, we may need to define it operationally more clearly, it may include capacity of the system to deliver and I think, it should be clearer in the framework. We need to consider it. If we include it, it will not be forgotten later while developing a guideline. The framework touches is somewhere but not emphasized enough. Its about policy execute-ability.<br>*Interviewer: What you want to is not just the legal aspect but also administrative and managerial aspects should be considered. Am I right?*<br>*P1: Yes, its somewhere but not explicit."*	*MAY enter your...*<br>*P2:Your enabling environment.*<br>*P1: Interviewer: I'm not here on active basis.*<br>*P3: For some countries.*<br>*P4: [...] I think actually in that space, feasibility and health systems (as he has mentioned), the „health systems" is government. And government is those things of governance and governing, itself. So that first bullet in there, needs to be beefed up to capture those aspects. Because you can have resistance just because of politics.*
	**Interaction with and impact on health system**	**• Reassess wording & definition // consider missing sub-criterion: dependency on activity & action of other elements of the health system as a basis for intervention implementation and functioning**<br>*" I don't know exactly if this is somewhere here, maybe, or maybe it isn't so linked, but there're some recommendations of ours, of the (), that for instance, involves a person with HIV, so we may even try to implement this recommendation, if the HIV and AIDS department doesn't adopt these recommendations too it isn't going to work, or for example, we want to define something for prisons, if the Ministry of Justice doesn't adopt it, it isn't going to work. So I don't know how it could be included, even because it isn't all the recommendations, that we need the*		**• Reassess wording & definition // consider missing sub-criterion:** Consider whether the criterion is adequately described and whether an expansion on the criterion is needed.<br>*I think, the matter of "integration" is missing. Nowhere in the framework it explores the aspects of integration (how and why interventions should be integrated or not). It should be captured somewhere*<br>**• Reassess wording & definition // consider missing sub-criterion:** Consider whether the criterion is adequately described to emphasize the broad understanding of health system and/or whether an aspect of intersectoral cooperation is missing.	**• Reassess wording & definition // consider missing sub-criterion:** Consider whether the criterion is adequately described to emphasize the broad understanding of health system and/or whether an aspect of intersectoral cooperation is missing.<br>*Number one, um, I don't know where exactly to fit it in, but I think it would be good to add some information on linkages with other sectors, like agriculture, industry, you know. In-in regard to-to issues of reuse and recycling*.

		partner a lot for it to happen, of the issue of feasibility, maybe specifying it here the feasibility when these partners are necessary, if it doesn't exist, it won't happen."		*"Interviewer: What you mean here is that when we talk about the context, it is not not just HF and health system but other system with whom we will be engaged as well. As P3: said, it will be for effective integration with other sector. P4: Yes. Where will that be captured? P3: I am thinking, may be we need to add a new category P4: Though there is a sub-category on feasibility. PS I think, it should fit there. We should try to. P5: Complex intervention requires oordination with other sectors. That requires clear consideration. Let us propose a new sub-criteria "*	
	**Need for, usage of and impact on health workforce and human resources**			• **Reassess wording & definition // consider missing sub-criterion:** Consider whether the criterion adequately reflects the heterogeneity of infrastructure availability on all links of the implementation chain of an intervention<br>*In the framework, the guideline is very comprehensive. But, I am not sure how this framework considers "availability of local resources". Though the guideline has "equity" as criteria, how do we consider service delivery in un-reached areas, in areas with and without road network, etc. We have tried different outreach models, eg, roving ANMs modal for example. When introducing such interventions, how do we consider if there are enough ANMs willing to function as we wish. "Do we have required human resources, required financial resources locally available etc" should be considered somewhere in the framework.*	

	**Need for, usage of and impact on infrastructure**	**• Reassess wording & definition // consider missing sub-criterion: availability and capacity of institutions and structures for planning, monitoring, and evaluation of intervention**:<br>*"P1: I think, I don't know if it fits well as a criterion, but I think we should think of a way to monitor, [...] P2.: A separate criterion for planning, monitoring, and evaluation, because then it includes a bit of what [Name] brought up, in this initial set, resources, feasibility which is already part of planning process anyway, so maybe a criterion. I also think I missed this integration process a little, that we could bring a little from a criterion that dialogued with this process, sort of this image that we've got here, in the front, that does something cyclic, that does a circular process really, of dialogic, of discussion, of bringing up these constructs more, a more integrated evidence… Let's say, a little more integrated, articulated, right?"*		**• Reassess wording & definition // consider missing sub-criterion:** Consider whether the criterion adequately reflects not only the issue of currently available infrastructure, but also the issue of availability, implementability and procurement of needed infrastructure<br>*"For example, in FP we have introduced EC as an option of FP methods. We didn't have clear idea how that will be procured (as that was not in the list of essential drugs/commodities of MOH). We used FHD funds to procure and sent that to service delivery sites, but most of that was expired as all EC users purchase it from private medical shops, they do not approach health facilities to get them. We should consider all these aspects too."*<br>**• Reassess wording & definition // consider missing sub-criterion:** Consider whether the criterion adequately reflects the heterogeneity of infrastructure availability on all links of the implementation chain of an intervention.<br>*We discussed earlier that school and health should coordinate at local level. We prepared guideline accordingly. We provided materials (IEC materials) for the school thinking that they will keep that in their library. They don't have shelf to keep those materials. They don't have space. (in many schools). They struggled to store and distribute those materials. They faced challenge to manage it. We didn't thought about it.*<br>**• Wording and definition // consider criterion reported as missing**: availability and capacity of institutions and structures	**• Wording and definition // consider criterion reported as missing**: availability and capacity of institutions and structures for planning, monitoring, and evaluation of intervention

				for planning, monitoring, and evaluation of intervention<br>*"I think we are missing out to monitoring guidelines. Where is it? When you implement, you produce guidelines, then people are implementing. How do you evaluate whether they're making an impact in the community? You need that all the users are using them properly and that (appearance). So we must have a framework, a monitoring and evaluation framework... for these guidelines."*	
**Evidence Considerations**	**General**				
**Missing (sub-)criteria**		**Consider missing criterion: Sustainability of intervention / recommendation**<br>*"P1: The issue of the sustainability of these recommendations, I don't know if it's been included, if it's been considered or not… P2: This is important, right? P3: Yes."*	• **Missing criterion or aspect:** consider, whether political considerations (eg, in the form of lobbyism should be considered more explicitly (eg, as part of a criterion of political feasibility).<br>*P1: Where would you position the word lobbyism?*<br>*Interviewer: [...] That was one of the political decisions we made [...] that political interests, in the sense of lobbying, do not come in as a relevant decision criterion, but should be considered under acceptability. There we had acceptability on public or other relevant stakeholders. Is that sufficient? Or would you say that lobbying or political considerations should simply be a conscious decision criterion?*<br>*B8: Yes, as you say, it is implied, not implied. It is just not so clear. But, as you say, it does express it.*<br>*B3: So, should lobbyists be in it or should one say: "This should not really play a role in a decision-making framework.*	• **Missing criterion or aspect:** consider, whether a criterion regarding multi-sectoral collaboration is adequately captured.<br>*P7: Multi-sectoral collaboration and their role should be captured somewhere, it is not just about infrastructure but also about roles RB As in mensuration hygiene, we need to collaborate with WASH, infrastructure, health etc GP While we develop a guideline, the guideline should reflect what roles are primary responsibilities of health sector and what roles are the responsibilities of other sector? This applies to other guidelines too, not just for adolescent health guidelines*	• **Missing criterion or aspect:** Consider whether the sustainability and life-course perspective regarding feasibility (maintenance, repairs etc.) is adequately reflected or if a new criterion should be added.<br>*Interviewer: Um, do you think it's applicable within your context, within the national context in Uganda?*<br>*P1: I think it's, it does, but I think something that was, something around technological [inaudible], something like that is missing. Uh, with the sanitation systems, much of it around also the cost of-of replacement. So, the issue around say composting is very important. So, if you're going to go ahead and take note of this then you have to bring in maybe another dimension around market, I think.*<br>• **Missing criterion or aspect:** Consider whether the issue of reliability and quality of an intervention is adequately covered in the framework or needs to be added.

			B2: The only question is: Is it allowed?<br>*B7: Well, you won't be able to prevent it from playing a role in the decision. But..."*		*I don't know where this would fit, but looking through the different criteria and really focusing on service provision. The different aspects that really qualify a service to be a service. Is that pit emptying, or the fecal sludge management aspects affordable? For example, uh, are they accessible? Are they reliable? [agreement from group] Um, because when you look at it from the service point of view, then you get into the SDG 6.3 that you read for us when we were starting. Is the quality of the service, uh, really visible? And-and people can really say, "we are ready to pay slightly more if the service is this"*
**Order & grouping of criteria in general**		**Reordering the criteria: Balance of health benefits and harms first, followed by "feasibility-oriented" criteria financial and economic considerations and health systems and feasibility considerations**<br>*"Thinking of, I mean, in practice, if we think, there's the recommendation, and now, what are we going to do about it? And using this instrument, I think this first topic here, to ponder risks and benefits, it really has to be the first, because if there's no benefit, we don't even discuss it, but I think these two last ones, which are financial and economic considerations that, I mean, they have to exist, otherwise there's no way, and considerations on feasibility, there's gotta be feasibility, otherwise there's no way. They'd have to come before. I don't see why we should discuss the rest if these previous three aren't ok. And these next ones, which are human rights, health equity, official education, they'll depend a lot on, in my*		• **Order & grouping of criteria:** Consider whether societal impact and "health impact" should be considered within one criterion or alongside with each other.<br>*P8: This societal impact is linked with other aspects as well. What if we merge societal impact with health impact as well?*<br>• **Order & grouping of criteria:** Consider whether "health systems considerations" should be moved to a higher position within the framework to emphasize the criterion's importance.<br>*P1: Health system consideration is very important. We never, esp not in MDG period thought about it. Now it SDG period this is being focused. People are talking about it. Now, in terms of orders, if we can keep this forward on the list, that would be better I think. Though the list is not on the order of the priority, this is taken in that way and sometime there is*	

		view, the way we're going to organize this policy, what the strategies are, what the criteria are, who is going to receive, who isn't, what the target audiences are. So I think they should come after the other three are ok, right?"		*a risk of missing points or less emphasis on the points that appear towards the end. It would be great if the order can be reconsidered. Interviewer: So, you would suggest the group to reconsider the position of health system consideration*	
