# Supplementary material for: Advancing the WHO-INTEGRATE Framework as a Tool for Evidence-Informed, Deliberative Decision-Making Processes: Exploring the Views of Developers and Users of WHO Guidelines
Source: Int J Health Policy Manag. 2020 Oct 27;11(5):629–41. doi: 10.34172/ijhpm.2020.193 (PMC9309924; doi:10.34172/ijhpm.2020.193)
Supplement: Supplementary file 6 — Coverage of Decision Aspects Stated in the FGDs by Criteria and Sub-criteria of the WHO-INTEGRATE Framework. [file ijhpm-11-629-s006.pdf]

Supplementary file 6. Coverage of Decision Aspects Stated in the Focus Group Discussions by Criteria and Sub-criteria of the WHO-INTEGRATE Framework

Supplement Table S6.1: Text examples of decision-making aspects and considerations discussed in the respective Focus Group Discussions (FGDs) in Brazil, Germany, Nepal and Uganda set in relation to those criteria and sub-criteria in the WHO-INTEGRATE Framework.

Sub-Criterion	FGD Brazil	FGD Germany	FGD Nepal	FGD Uganda
**Balance of health benefits and harms**				
**Efficacy or effectiveness on health of individuals**	„I found it nice that you separated individual and population. It's because I see that, in our case TB is sensitive, we focus on the population a lot, but when we realize they are very resistant, it's a few cases, and treatments are expensive, so we have to look at the individual, not to forget this population, so I found it nice to separate."		„The benefits as we define as public health and medical professional[s] and the benefits that adolescent would define in that age is different. The pleasure of being together with a partner, physical contacts, enjoying beer and cigarette for them is special. I am not sure if that benefits is considered [..] here […]."	„Because if you don't manage it [wastewater, sludge], the repercussions are critical. It can be source of diseases. It can bring, uh, uncomforted, can smell. Because even if it's not next to me, it can give me infection. I wouldn't want the smell to go over my fence and reach me. It is also good human practice any way. [This] is unhealthy."
**Effectiveness or impact on health of population**	„I found it nice that you separated individual and population. It's because I see that, in our [work on TB], we focus on the population a lot, but when we realize they are very resistant, it's a few cases, and treatments are expensive, so we have to look at the individual, not to forget this population, so I found it nice to separate."	„[..] because in principle one can say nothing [about whether or not isoglucose is harmful or not], except we do not know. Yes, if you take a look at the literature, nothing comes out, it goes out like the Hornberger shooting."	„The benefits as we define as public health and medical professional[s] and the benefits that adolescent would define in that age is different. The pleasure of being together with a partner, physical contacts, enjoying beer and cigarette for them is special. I am not sure if that benefits is considered [..] here […]."	„it will help community live in a clean and hygienic environment, if it safely managed. It will reduce on the transmission of infectious diseases, especially like the sanitation and hygiene related: the cholera, the typhoid, the hepatitis E, the dysenteries, the diarrheas. It will reduce on that."
**Patients'/beneficiaries' values in relation to health outcomes**		„It's almost like smoking. I mean that smoking is harmful to health. I don't think we need to discuss it. Nevertheless, I do not want to ban myself from smoking. I don't smoke, I don't smoke anymore. Nevertheless, I do not want to be banned from smoking by law. That is perhaps a very hard example. I like to drink wine and alcohol regularly. This discussion also raises the fact that alcohol is harmful to health. I do not think we need to discuss it here. But I do not want to be banned from categorically no longer drinking alcohol."	„The benefits as we define as public health and medical professional and the benefits that adolescent would define in that age is different. The pleasure of being together with a partner, physical contacts, enjoying beer and cigarette for them is special. I am not sure if that benefits is considered and if here or somewhere else"	
**Safety-risk-profile of**	„So, despite all [...] Health risks, sometimes it's considered a bit better than leaving the patient			

**intervention**	with no treatment at all, so sometimes it isn't even this risk and benefit analysis, but it's as if it were the only alternative. We approach many medications like that, unfortunately."			
**Broader positive or negative health-related impacts**			„We feel now that we have done some mistakes then. When we interacted with adolescents, we realized that they are more vulnerable now as a result of dedicated services space and dates, as their parents would think that they must have done something wrong, so are they going to these clinics. It stigmatized the services. We felt it now."	„Okay, because if you don't [manage wastewater adequately], especially in rural areas, it becomes a breeding ground for mosquitoes."
**Human rights and socio-cultural acceptability**				
**Accordance with universal human rights standards**	„I don't know if it's pertinent, but I have the impression one of the criteria was the dignity of people with TB, even because one of the main axes is the person-centered treatment."			„[Reasons for opposing an intervention or guideline recommendation] [..] If [they] are not user friendly, if they are impinging on human rights, if they are [..] discriminative, not bring everyone onboard, dictator, if the guiders are dictating terms."
**Socio-cultural acceptability of intervention to patients/ beneficiaries and those implementing the intervention**	„[…] we realized because of some previous knowledge which the lab technicians had, […], they sometimes received that new equipment with a certain suspicion, so instead of replacing the [..] test, they did both, and it ended up generating more work for them, so the socio-cultural acceptability would be in that sense too ()."		P1: „So, when we decide, we need to consider community acceptability and availability of resources. These are important in making decisions while preparing a guideline."<br>P2: „We wanted to orient school teachers in Gorkha district on Family Planning, but no one wanted to come. As some of the schools in the districts are being merged into others due to limited students, they had a fear that their job will be insecure if there are lesser number of children in future. We never considered those kind of societal impact."	„You must respect peoples' freedom, norms, cultures and religions as she has said."
**Socio-cultural acceptability of intervention to the public and other relevant stakeholder**	„But we, here the criterion doesn't show, the intervention of patient beneficiaries, so it may even be, it isn't the patient directly but the people who are there around him or her and those who implement the intervention, […]"	„In this situation we are perhaps not in the same situation as in the executive area, where I must have very clear evidence that stands up in court. But I must at least have legitimacy, which can also be sociological. So what do I know? The people in my country do not want genetic engineering. The people in my country	„Cultural and ethical aspects are address very well and carefully. That has been the focus."	

**groups**		do not want steroidal fattening additives-“		
**Impact on autonomy of concerned stakeholders**	„[..] to what extent does this possibility of acceptance go considering that we can’t measure the knowledge they have on the subject to oppose it […]? So, it’s everyone’s right in the health system to have the care that he or she considers the best one. But to what extent does this limit of divergence go when […] [a patient holds a specific belief] [but] all the evidence and scientific studies and organizations point to the contrary. […] So I don’t know if there is a limit up to which you consider this or you don’t.”	„But I want to remain a responsible consumer, and I do not want to be banned on the basis of such evidence.“	„Informed consent is important esp for the family planning services. We need to focus on counselling, we need to let them decide their choice.“	„[Reasons for opposing an intervention or guideline recommendation] „[..] If [they] are not user friendly, if they are impinging on human rights, if they are [..] discriminative, not bring everyone onboard, dictator, if the guiders are dictating terms. […] It is not gender, it is not eh inclusive.”
**Intrusiveness of intervention**		„This ban goes relatively far. And in order to legitimise something like this, I believe that far-reaching evidence is also necessary to legitimise the meaning of this. And not just an assumption. The less far-reaching the intervention, the less sufficient legitimacy I need. That means demanding labelling as long as it is neutral [...]. Yes, and then we can still try to clarify the matter. That means that the state must, in my opinion, exploit its repertoire in different ways. And that's what's going on here right now, and the danger situation is unclear at best.“		„You must respect peoples’ freedom, norms, cultures and religions as she has said.“
**Equity, Equality and Non-Discrimination**				
**Impact on health equality and/or health equity**		„Because, especially if you always go on this information and education track, you know very well that different social groups then have different needs, can be taken up and implemented differently. And I think it is important for us too. And we have enough evidence, actually. So in general we should try to reduce our preventive efforts for health, not to increase social inequalities, but rather to reduce them. In this respect, it is also important for us to give priority to measures that do not cement social inequality even		[Reasons for opposing an intervention or guideline recommendation] „Leaving some people behind, then it is most likely to meet a stiff rejection.“

		more, as it already is. Well, I actually think that this is an important argument, even for us.“		
**Distribution of benefits and harms of intervention**	„At the beginning we were palling [..] who would have access to the test, there were many possibilities, for instance, [...] let’s offer to vulnerable populations only, certain populations, and it fell through quickly, because I didn’t have, I couldn’t justify it,. [...] If we design a policy that somehow it affects someone’s rights, it’s almost immediate that someone, some organization or some person will notice that and chase us up for a change in direction.“	„And there is only one thing I see as alarming: I think you make it a bit easy for yourself when you then say that we can't solve it any other way, we now simply inform the people who should decide. [...] And we already know that half of the population in Germany does not have sufficient health competence. And I mean, if we can't agree on that, is that good or bad now? The consumer on the street then certainly not? So I think we just make it easy for ourselves.“		„You must put into consideration that it does not expose even the people working into getting infection when they are in that process of managing the fecal sludge. You must protect and preserve people’s health, human health.“
**Affordability of intervention**	„[…] I’d mention the financial issue, financial, economic for several reasons, even for you to put stuff in the system you’ve gotta evaluate the cost-benefit, several types of costs, there are the catastrophic costs now that we’re bringing the patient too that thinks of the costs, on every side, so every time for a decision process I’d bring it to our reality too, which is considering the question, that I’d bring something like equity, equality a lot of cost-related stuff gets here too for us to make things equal.“		„We also need to consider how the users are going to get these commodities and what access and cost implications for them are“.	„If you are going to roll them out and they are going to impose huge costs on countries or individuals, uh people may reject them.“
**Accessibility of intervention**	„We define the places to receive the equipment of the rapid molecular test, we consider all the regions equally as for the epidemiological and operational restrictions, we would be thinking of the right of access to everybody and the socio-cultural acceptability“	„And there is only one thing I see as alarming: I think you make it a bit easy for yourself when you then say that we can't solve it any other way, we now simply inform the people who should decide. [...] And we already know that half of the population in Germany does not have sufficient health competence. And I mean, if we can't agree on that, is that good or bad now? The consumer on the street then certainly not? So I think we just make it easy for ourselves.“	„We also consider practical aspects. As we know that most of the adolescents are school going, they are in the schools most of the time. Therefore, while deciding where to set-up an adolescent friendly service center (clinic), we prioritized health facilities near to a school so that it is easier for adolescent to seek services from there.“	„If the guideline [recommendations] are also too technical and the teams that are proposing those guidelines have not taken the time to break it down. If it remains so scientific, for example, my grandmother would never understand such things, [..] then it prevents even any kind of welcoming because you have started with the too high technical, too complex.“

**Severity and/or rarity of the condition**		P1: „For example, we recall BSE [Bovine spongiforme Enzephalopathie] in 2000, where there were no studies whatsoever and a completely new outbreak of a disease and where, as a preventive consumer protection measure, very violent and massive steps were taken […]. And there nothing came out up to today fortunately [.]. But massive action has also been taken. And did not say, one needs ten years or five studies or so […] And in the case of BSE, of course, the suspected dangerous situation was so serious that one could and probably had to take action beforehand.”<br>P2: „And how serious that is. Yes, that is what I mean, the whole proportionality test that has to take place.“		
**Lack of a suitable alternative**	„So, despite all [...] Health risks, sometimes it’s considered a bit better than leaving the patient with no treatment at all, so sometimes it isn’t even this risk and benefit analysis, but it’s as if it were the only alternative. We approach many medications like that, unfortunately.“	„Yes, and in the case of BSE [Bovine spongiforme Enzephalopathie] there was no other way of protecting the consumer [...]. You do not have to try to prohibit isoglucose. You get the opportunity to clarify, to declare and so on. So that is a little bit, because we do not have to take such radical measures.“		
**Societal Implications**				
**Social impact**			„We wanted to orient school teachers in Gorkha district on Family Planning, but no one wanted to come. As some of the schools in the districts are being merged into others due to limited students, they had a fear that their job will be insecure if there are lesser number of children in future. We never considered those kind of societal impact.“	
**Environmental**				„Systems should not pollute their environment

**impact**				instead of protecting the environment.“
**Financial and Economic Considerations**				
**General**	„[..] financial and economic considerations, they’re really very oriented to the beginning of the planning, and then to the sustainability of what you’re planning, I think the economic impact on the patient’s life, it can be included as a justification for your managers, for you to convince them that the strategy is important. […] the molecular rapid test, for instance, we can’t implement [it] in all the municipalities and it wouldn’t make sense to do that, it’s too expensive for us to put it in low-load munici-palities, where the equipment is going to get idle, we did this type of evaluation before.“			„If you don’t manage it, if you don’t put money into it, if you don’t plan on how to manage it, this is what you will suffer from as a community, as a household, as a country. And then doing a cost-benefit analysis, or what-what are the losses and-and-and gains, so that that is clear.“
**Financial impact**	„[…] I’d mention the financial issue, financial, economic for several reasons, even for you to put stuff in the system you’ve gotta evaluate the cost-benefit, several types of costs, there are the catastrophic costs now that we’re bringing the patient too that thinks of the costs, on every side, so every time for a decision process I’d bring it to our reality too, […], that I’d bring something like equity, equality a lot of cost-related stuff gets here too for us to make things equal.“		„So, when we decide, we need to consider community acceptability and availability of resources. These are important in making decisions while preparing a guideline.“ […] „Economical is the aspect that we haven’t considered enough. We haven’t been able to address such matters while establishing adolescent clinics as well.“	„If [..] the cost is prohibitive, then it will not happen.“
**Impact on economy**		„Yes, I think that is the second possibility, that one does not commit oneself to the health rail [argument for action against Isoglucose based on health reasons], but to the different interests, let's say now and see this from the economic side. So of course that can also be an argument.“		„I think, that is impact on the economy. It would help linking economies, to develop, because people would be rid of sicknesses which are sanitation- and hygiene-related, so they must make a saving, and contribute economically to that particular country.“
**Ratio of costs**	„[…] I’d mention the financial issue, financial,			„If you don’t manage it, if you don’t put money

**and benefits**	economic for several reasons, even for you to put stuff in the system you've gotta evaluate the cost-benefit, several types of costs, there are the catastrophic costs now that we're bringing the patient too that thinks of the costs, on every side, so every time for a decision process I'd bring it to our reality too, […], that I'd bring something like equity, equality a lot of cost-related stuff gets here too for us to make things equal.“			into it, if you don't plan on how to manage it, this is what you will suffer from as a community, as a household, as a country. And then doing a cost-benefit analysis, or what-what are the losses and-and-and gains, so that that is clear.“
**Feasibility and Health System considerations**				
**General**	„I agree [..] that both the financial question and [that][..] technical feasibility, considering the feasibility important because I can't guarantee either equity or equality if I can't get the necessary partners to make that possible, first,“			„If somebody feels that maybe the guideline [recommendation] is so high, the standard is so high that it cannot be achieved, or it would actually claim a significant amount of their resources, then they would want to think that it is prohibitive.“
**Legislation**	„[…] because most medicines have no registry in the country, and this is a problem for us, but we've tried to work thinking that TB is still a disease that doesn't have many alternatives, so those available, we try to bring here to improve this treatment. Just to resume a few things.“	„[…] [Whether] a differentiated labelling obligation of isoglucose on food […] is legally possible at all, one would have to consider that.“	„In my view, we need to consider what the national laws say, while developing a guideline. For example, in our law especially the Muluki Ain [civil code law], any sexual intercourse with a person of less than 16 years is considered as a rape and any marriage below the age of 20 is considered as child marriage. Therefore, we also considered national law while preparing a program guideline.“	„it will help community live in a clean and hygienic environment, if it safely managed. […] It's good to live in a clean and health environment, as stated by Constitution of Uganda that every human being, everyone is entitled to clean and health environment (Article 17). So, I think it is very important to manage waste properly in this country.“
**Leadership and governance**		„There's Trump's duties, for example. An intervention of the same caliber [in comparison with restriction of isoglucose imports]. And they do not consider the questioning of this activity by World Trade Court. Because he says:“I don't care, because I am authorized by the sovereign to do so.“		„[..] Do ALL countries have regulatory systems to regulate? Where these systems are not working well. We know not all governments have systems. I was in [country], they have no government! They are run by NGOs, the WHOLE government. So now, you see, there must be legal systems to counteract systems which are in the guidelines, because they are sanitation guidelines, because we have systems there, but safeguards by countries? Safeguard these guidelines, that they are not going beyond what the country has prescribed in their laws.“
**Interaction with and impact on**	„There is a wide variety of actions, strategies, that they have to select, taking into		„We need to assess if new guideline helps the process of operationalization and HRH	

**health system**	consideration even their work capacity, so sometimes in a municipality, a state, there's a well-established, well-consolidated HIV program, it can work the TB-HIV questions well, but can't work TBS questions, so they can organize their structure according to what is already consolidated and then try to improve what is recommended that will cause them to have more work later."		dynamics. We need to asses if it does affect regular operation of health system. For example, in CB NCP program FCHVs were trained for many new functions and as a result, they forgot their core health promotion role. That was diluted."	
**Need for, usage of and impact on health workforce and human resources**	„[…] we realized because of some previous knowledge [..] the lab technicians […] sometimes received that new equipment with a certain suspicion, so instead of replacing the [..] test, they did both, and it ended up generating more work for them […]."		„Interviewer: [...] In context of Nepal, while deciding right interventions to deliver family planning, adolescent and reproductive health services, what are the considerations and what do you think would play important role?<br>P1: I think, commodities, provider and if they are trained or not, capacity of those providers, type of facility and its infrastructure, ability to maintain privacy, etc."	
**Need for, usage of and impact on infrastructure**			„We provided materials (IEC materials) for the school thinking that they will keep that in their library. They don't have shelf to keep those materials. They don't have space. (in many schools). They struggled to store and distribute those materials. They faced challenge to manage it. We didn't thought about it."	„If somebody feels that maybe the guideline [recommendation] is so high, the standard is so high that it cannot be achieved, or it would actually claim a significant amount of their resources, then they would want to think that it is prohibitive."<br>[The criterion uses a broad concept of infrastructure, see WHO-INTEGRATE framework for details]
**Evidence Considerations**				

**General**	„[…] the question of efficacy is complex, I'm thinking of us here, how to evaluate this. Effectiveness may be a little easier, but the impact may be, so, thinking of how the Ministry of Health is complex, to think how to do this in the side of the chain.“<br>„We're looking for another department; we've expanded our conversation with the people from the HIV and AIDS treatment […]. […] So I think the interesting point is that scientific evidence got in, and it's being used, it's already being an example when we go to a seminar, we can say“look, talk to your depart…, with the other departments, we're doing this, this way, and it's working, or it isn't working“.	„Is a state legitimated to act even when there is no scientific evidence? Or is a state legitimated not to act when scientific evidence shows the need for action? And in this dilemma or at this point one finds oneself in the situation. Because it is by no means so that we know - and that is why isoglucose example is wonderfully suitable - because in principle one can say nothing, except we do not know. Yes, if you take a look at the literature, nothing comes out, it goes out like the Hornberger shooting. And against this background it is actually not ready for scientific decision.“	„As [Interviewer] said earlier, even the quality of evidence differs. Some evidence are generated from small scale study, others are based on systematic reviews. I think, guidelines are developed by appraising all these evidences and thus are useful for us.“	„If you are saying that if I don't take care of the faecal sludge, it will develop into this, you must back it with scientific evidence, with the data, to prove that what you are saying is correct.“
